# Positive selection in dNTPase SAMHD1 throughout mammalian evolution

**DOI:** 10.1073/pnas.1908755116

**Published:** 2019-08-26

**Authors:** Christopher Monit, Elizabeth R. Morris, Christopher Ruis, Bart Szafran, Grant Thiltgen, Ming-Han Chloe Tsai, N. Avrion Mitchison, Kate N. Bishop, Jonathan P. Stoye, Ian A. Taylor, Ariberto Fassati, Richard A. Goldstein

**Affiliations:** ^a^Division of Infection and Immunity, University College London, WC1E 6BT London, United Kingdom;; ^b^Macromolecular Structure Laboratory, The Francis Crick Institute, NW1 1AT London, United Kingdom;; ^c^Retrovirus–Host Interactions Laboratory, The Francis Crick Institute, NW1 1AT London, United Kingdom;; ^d^Retroviral Replication Laboratory, The Francis Crick Institute, NW1 1AT London, United Kingdom

**Keywords:** SAMHD1, HIV-1, restriction, evolution, mammals

## Abstract

The vertebrate protein SAMHD1 is highly unusual in having roles in cellular metabolic regulation, antiviral restriction, and regulation of innate immunity. Its deoxynucleoside triphosphohydrolase activity regulates cellular dNTP concentration, reducing levels below those required by lentiviruses and other viruses to replicate. To counter this threat, some primate lentiviruses encode accessory proteins that bind SAMHD1 and induce its degradation; in turn, positive diversifying selection has been observed in regions bound by these lentiviral proteins, suggesting that primate SAMHD1 has coevolved to evade these countermeasures. Moreover, deleterious polymorphisms in human *SAMHD1* are associated with autoimmune disease linked to uncontrolled DNA synthesis of endogenous retroelements. Little is known about how evolutionary pressures affect these different SAMHD1 functions. Here, we examine the deeper history of these interactions by testing whether evolutionary signatures in SAMHD1 extend to other mammalian groups and exploring the molecular basis of this coevolution. Using codon-based likelihood models, we find positive selection in SAMHD1 within each mammal lineage for which sequence data are available. We observe positive selection at sites clustered around T592, a residue that is phosphorylated to regulate SAMHD1 activity. We verify experimentally that mutations within this cluster affect catalytic rate and lentiviral restriction, suggesting that virus–host coevolution has required adaptations of enzymatic function. Thus, persistent positive selection may have involved the adaptation of SAMHD1 regulation to balance antiviral, metabolic, and innate immunity functions.

## Significance

Animals defend themselves from viral infection using innate immunity proteins that disrupt various stages of the virus life cycle. In response, viruses produce proteins that bind these host factors and compromise their activity, resulting in evolutionary conflict as immunity and virus proteins adapt to prevent and restore binding, respectively. We report that evolutionary conflict involving the host innate immunity protein SAMHD1 has occurred throughout mammalian evolution. We observe adaptation in a region of SAMHD1 that regulates its activity, and we demonstrate how mutations here influence its enzymatic properties, suggesting that evolutionary conflict has involved modulation of SAMHD1 regulation and function. This correlates with reduced restriction of HIV-1, indicating that positive selection has influenced both SAMHD1’s dNTPase and antiviral activities.

The parasitic nature of their lifestyle brings viruses into evolutionary conflict with the immune systems of their hosts. Vertebrates have evolved an arsenal of innate immunity proteins, called restriction factors, that target conserved features of virus replication cycles, while some viruses, in turn, have evolved means of neutralizing (or “antagonizing”) them, often by mechanisms involving direct protein–protein interactions (1, 2). This leads to an evolutionary “arms race” as the restriction factor undergoes rapid evolution to alter the interaction interface and prevent recognition by a viral antagonist, while the antagonist similarly evolves to restore binding.

SAMHD1 (sterile alpha motif and histidine-aspartic acid domain-containing protein 1) is a restriction factor of several groups of retroviruses and DNA viruses, including lentiviruses [namely, HIV, simian immunodeficiency virus (SIV), and feline immunodeficiency virus (FIV)], vaccinia, herpes simplex 1, and hepatitis B viruses (3–10). Its deoxynucleoside triphosphohydrolase (dNTP-tpase) activity suppresses viral replication by hydrolyzing dNTPs, reducing the intracellular concentration of substrates required for viral DNA production (11, 12). HIV-2 and related SIVs counter SAMHD1 by expressing the accessory protein Vpx that recruits SAMHD1 to DCAF1, targeting it for degradation through the cellular Cullin-4–based E3 ubiquitin ligase machinery (3, 4, 13–16). Some other primate lentiviruses use the related Vpr protein to fulfill the same role (17), although HIV-1 Vpr does not have the equivalent function. Vpx/Vpr from different lentivirus lineages target different regions of SAMHD1, recognizing either the N or C termini (18). Evolutionary analyses of primate SAMHD1 have shown that positive diversifying selection has occurred in these 2 different binding regions, suggesting an evolutionary arms race between viruses and SAMHD1 in primates (17, 19). SAMHD1 antagonism by primate lentiviruses is often strikingly host-specific, including adaptation to dominant SAMHD1 alleles within species, suggesting that the evolutionary conflict has led to highly intricate coevolution (20).

In addition to its antiviral function, SAMHD1 also maintains the fine balance of intracellular dNTP levels that allows progression of the cell cycle (21), while preventing the accumulation of endogenous nucleic acids (22). The enzyme’s activity is regulated by conversion between the catalytically active tetrameric state and the weakly active monomeric or dimeric forms (23). Tetramers are favored in the presence of SAMHD1’s allosteric regulators, dNTP and GTP/dGTP molecules (24, 25), while phosphorylation of threonine residue 592 (T592), located near the C terminus, reduces the stability of the SAMHD1 tetramer, favoring the monomeric state. In both primates and mice, phosphorylation is mediated by CDKs 1/2 complexed with cyclin A2, suggesting that this mechanism of regulation is conserved among mammals (26–30).

Two crucial features of this molecular arms race remain unclear. First, since SAMHD1 is found throughout vertebrates, and DNA-producing viruses infect all domains of life, how widespread is the evolutionary conflict between viruses and SAMHD1 in other taxa? Second, how has SAMHD1 responded to selective pressure from its dual roles in virus restriction and dNTP regulation?

To address these questions, we applied codon-based likelihood models to a large set of SAMHD1 sequences from a diverse range of mammals. We found evidence of positive diversifying selection in every group of mammals for which data are available, indicating a pathogen–SAMHD1 arms race extending throughout mammalian evolution. Strikingly, many of the sites under positive selection cluster around T592, indicating positive selection acting on sites that modulate SAMHD1 phosphorylation, tetramerization, and, therefore, enzymatic activation. We show that replacing amino acids at some of these sites with residues observed in other mammal species reduces dNTP-tpase activity and can reduce HIV-1 restriction in cell culture. SAMHD1 has therefore experienced an unusual combination of selective constraints as selection pressure imposed by viruses interacted with the need to maintain, regulate, and adjust enzymatic activity.

## Results

### Positive Selection in Mammals.

To investigate the history of SAMHD1 during mammalian evolution, we compiled a dataset of 120 publicly available mammalian SAMHD1 coding sequences (*SI Appendix*, Table S1), including 5 well-represented clades: the Primates (*n* = 55), the Glires (rodents, rabbits, and hares; *n* = 16), the Cetartiodactyla (whales and even-toed ungulates; *n* = 18), the Carnivora (cats, dogs, bears, etc.; *n* = 8), and the Chiroptera (bats; *n* = 6). A phylogenetic tree estimated from these gene sequences by maximum likelihood was mostly concordant with the reported mammalian species phylogeny (31), and the majority of nodes had support values above 70% (*SI Appendix*, Fig. S1).

**Fig. 1.**
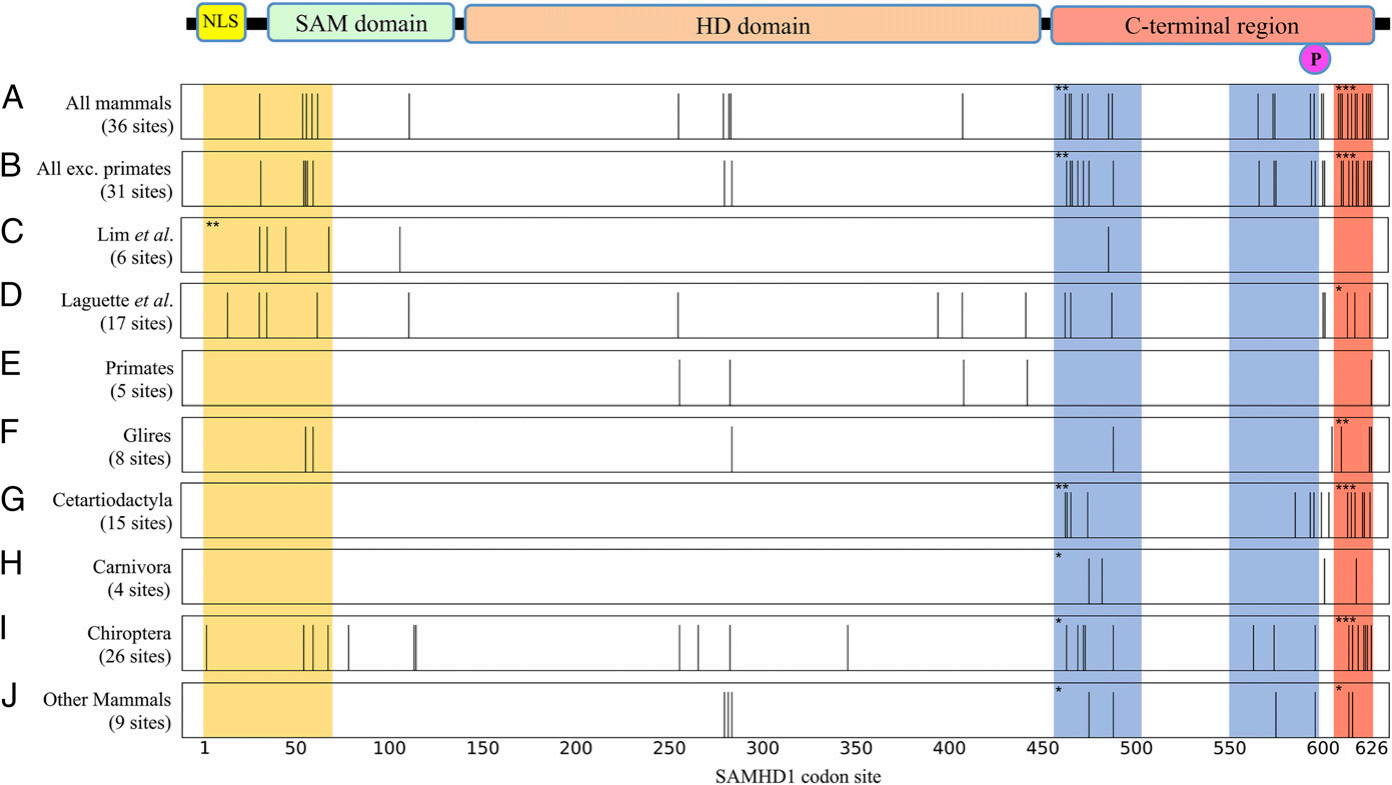
Codon sites under positive selection in mammalian SAMHD1 identified in this and previous studies. Panels represent the linear sequence, and vertical bars mark sites where the posterior probability of positive selection (Bayes empirical Bayes calculation) is greater than 0.95 (bar height is not meaningful). In parentheses are the total numbers of sites thus identified. Site numbering is based on the human sequence. At the top is a cartoon showing SAMHD1 sequence features. NLS, nuclear localization signal; P, phosphorylation site T592. Shaded regions correspond to the N-terminal Vpx/Vpr binding site (yellow), C-terminal Vpx/Vpr binding site (red), and region around the T592 phosphorylation site (blue), determined with reference to SAMHD1 crystal structures. Asterisks indicate where there is statistically significant clustering of sites identified as under positive selection in these sequence regions (*SI Appendix*, Table S5). *P < 0.05; **P < 0.01; ***P < 0.001. Note that the 2 sections comprising the phosphorylation region (blue) are treated as a single region. (*A*) Results from analysis of all mammals with PAML model M2a. (*B*) Similar analysis of mammals with primates excluded. (*C* and *D*) Published results of Lim et al. (17) and Laguette et al. (19), both from analyses of primate SAMHD1 with PAML model M8. (*E*–*J*) Sites identified in mammal subgroups, model M8.

Using the site-specific selection models implemented in PAML (32–34), we found that the likelihood-ratio test supported the presence of positive selection in mammalian SAMHD1 (*P* = 4 × 10^−90^; *SI Appendix*, Table S2), and 36 sites were identified as under positive selection (posterior probability > 0.95; Fig. 1*A* and *SI Appendix*, Table S3). We identified most of the same set of sites as under positive selection when repeating the analysis using 3 alternative tree topologies (*SI Appendix*, Tables S2 and S4), indicating that the result is not sensitive to possible minor inaccuracies in the estimated phylogeny topology.

As positive selection has been detected in primate SAMHD1 (17, 19), we sought to test whether this signature of a molecular arms race was specific to primates by repeating the analysis with the primate clade removed. Again, we found statistical support for positive selection (*P =* 3 × 10^−66^), and 31 sites were identified as under positive selection in nonprimate mammals (Fig. 1*B* and *SI Appendix*, Table S3). Of these, 29 had also been identified in the analysis of the all-mammals dataset (*SI Appendix*, Fig. S2*A*). These results indicate that positive selection is not confined to primate SAMHD1, but has also occurred in other mammals.

### Positive Selection in Mammal Subgroups.

We next determined whether the positive-selection signal was due to specific groups within mammals by repeating the analyses with each of the well-represented subclades. We also performed the analysis on a 6th group comprising the other species not belonging to a well-represented monophyletic set (*n* = 17), hereafter called “Other Mammals.” We observed evidence for positive selection in all of these subgroups. The identified sites for each subgroup were distributed in similar regions as sites found in our analysis of all mammals (Fig. 1 and *SI Appendix*, Table S3), although many of the identified sites were specific to a particular clade (*SI Appendix*, Fig. S2*B*). The Chiroptera (bats) had the greatest preponderance of sites, with 26 identified, while the Carnivora (dogs, cats, etc.) had the fewest sites, 4, all in the C-terminal region. These data indicate that positive selection has occurred in multiple groups throughout mammalian evolution. Only 5 sites were identified in the Primates (Fig. 1*E*), 4 of which were found by Laguette et al. (19) in their analyses of primate SAMHD1 using the same PAML models, while none of these were identified by Lim et al. (17) in a similar analysis, both presumably because of the larger number of sequences now available to us (35). Several of the sites reported by these authors as having posterior probabilities for positive selection above the conventional 0.95 threshold nonetheless had probabilities >0.90 in our analysis (*SI Appendix*, Fig. S3).

### Positive Selection at Vpx/Vpr Binding Regions.

We next examined whether the sites under positive selection in mammalian SAMHD1 are in the 2 distinct regions of the protein targeted by known SAMHD1 antagonists. We therefore mapped the identified sites onto crystal structures of primate SAMHD1 bound to lentiviral Vpx/Vpr proteins. We found 11 sites under positive selection in all mammals within the C-terminal Vpx/Vpr binding region (sites 606–626; ref. 36; *SI Appendix*, Fig. S4*A*), 5 of which have side chains directly contacting Vpx residues (sites 609; 610; 611; 618; and 622). By calculating the probability that 11 of 36 identified sites should fall in this binding region comprising 20 sites of a total 626 sites by chance (*Methods*), we found the concentration of sites identified as under positive selection in this region to be highly significant (*P* < 10^−6^). All but 1 of these Vpx-contacting sites (site 609) were also identified when primate sequences were excluded from the analysis, and the clustering of sites under positive selection in this region (calculated by the same approach) was also significant for sites identified in Glires, Cetartiodactyla, Chiroptera, and Other Mammals alone. We also identified several sites (32; 55; 57; 60; and 63) under positive selection across mammals in the N-terminal Vpx/Vpr binding region (sites 1–69; ref. 37; *SI Appendix*, Fig. S4*B*), where positive selection was similarly identified by Laguette et al. (19) and Lim et al. (17). This included sites in direct contact with Vpx, though this clustering was not statistically significant. Again, all but 1 of these (site 63) were also identified with primate sequences excluded. This overlap of sites under positive selection in mammals with regions bound by lentiviral Vpx/Vpr proteins, particularly the C-terminal binding site, suggests the existence of factors expressed by other viruses that may target similar regions of SAMHD1 in species other than primates.

### Positive Selection in C-Terminal Region Around Phosphorylation Site T592.

**Fig. 2.**
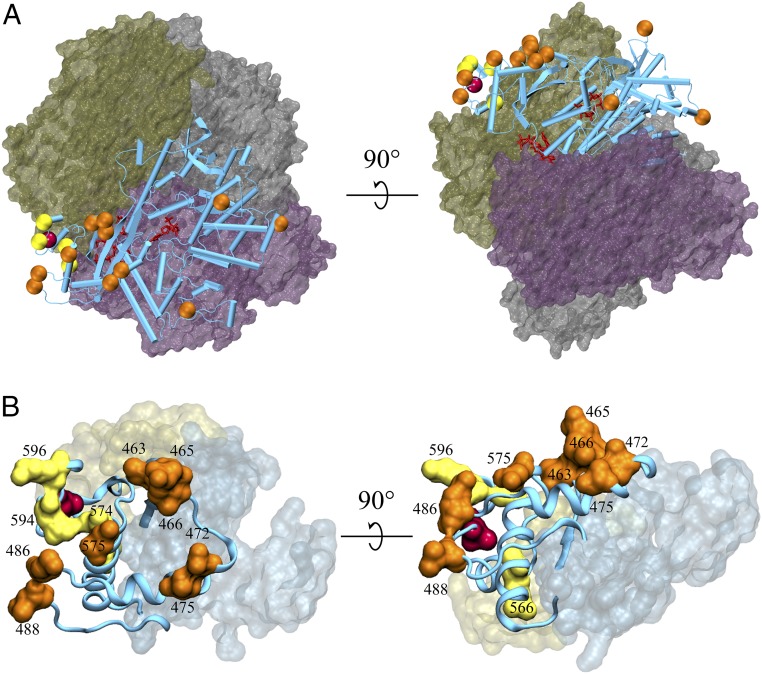
Sites identified as under positive selection in mammalian SAMHD1, shown on a published crystal structure (Protein Data Bank ID code 4TNP; ref. 25; residues 114–276 and 282–599). (*A*) Human SAMHD1 tetramer with foreground monomer shown as cyan cylindrical-helix cartoon and background monomers shown as colored surface representations. The 15 sites both under positive selection and present in this structure have their α-carbons shown as orange or yellow spheres, where yellow indicates sites in the C-terminal region chosen for point mutation experiments; threonine 592 is similarly shown and colored red. dNTP molecules bound in the foreground monomer are shown as red sticks. (*B*) Enlarged view of the C-terminal region, with foreground monomer shown as a plain cartoon, all atoms of sites under positive selection shown by surface representation, and background monomers as translucent surfaces. Highlighted sites are annotated with human sequence numbering, and site colors are as in *A*. (*B*, *Left*) Site 566 is obscured by site 575. (*B*, *Right*) Sites 574 and 594 are obscured by sites 575 and 486, respectively.

We observed clustering of sites under positive selection in 2 stretches of the C-terminal region (sites 456–502 and 550–599) that fold together to form a domain containing the phosphorylation site, T592 (Figs. 1 and 2). This clustering was statistically significant (*P* < 0.05) in all mammals, the Cetartiodactyla (whales and even-toed ungulates), the Chiroptera (bats), and the Other Mammals (*SI Appendix*, Table S5). None of the identified sites were located at the interface between SAMHD1 monomers or at the dNTP-binding catalytic or allosteric sites (Fig. 2*A*). The positioning of sites under positive selection in the region around the phosphorylation site therefore suggests that rapid evolution involved modulation of SAMHD1 function.

### Point Mutations at C-Terminal Sites under Positive Selection Affect SAMHD1 Function.

Given the interesting distribution of sites under positive selection, we next investigated the sensitivity of SAMHD1 structure, function, and regulation to replacements at positions under positive selection surrounding the phosphorylation site. We identified 4 sites of particular interest due to their structural context, proximity to T592, and the distribution of residues seen in different species (Fig. 2**,** yellow). Site 566 (arginine in humans) has undergone many physicochemically nonconservative substitutions to residues greatly varying in size, such as glycine, glutamine, and histidine, despite being mostly buried (*SI Appendix*, Fig. S5). Site 574 (alanine in humans) is almost entirely buried in the C-terminal region, yet residues with much larger side chains and contrasting physicochemical properties were observed in other species, including leucine, phenylalanine, and serine (*SI Appendix*, Fig. S6). Sites 594 and 596 (glutamine and lysine in humans, respectively) are both very close to T592 (Fig. 2*B*), and, again, chemically diverse residues were observed at these positions (*SI Appendix*, Figs. S7 and S8). The amino acid at site 596 marks the divergence of New World monkeys (negatively charged aspartate) from Old World monkeys and apes (positively charged lysine). To investigate whether these residues are likely to have the same structural context across species, we used homology modeling to predict animal SAMHD1 protein structures (38). Comparing each animal model structure to the human structure, the minimum root mean square deviation (rmsd) of atoms was below 0.4 Å, and corresponding Z-scores with respect to random structure alignments were all above 50, indicating very close structural similarity (*SI Appendix*, Table S6; ref. 39).

We then selected 1 or more residues that had been observed in multiple animal species at these 4 sites and introduced them into a human SAMHD1 background to test effects on function. Further homology-modeling experiments had shown that these residues had almost identical position and orientation when comparing mutant human SAMHD1 model structures and animal model structures for which these residues are wild type (WT), as measured by rmsd between atoms in their side chains; this suggested that the mutant residues in the human background accurately recapitulate their situation in their respective WT animal SAMHD1 structures (*SI Appendix*, Table S7 and Fig. S9). WT and mutant SAMHD1 constructs were expressed in *Escherichia coli*, and the activator and substrate dependence of tetramerization of purified proteins was analyzed by size-exclusion chromatography coupled to multiangle laser light scattering (SEC-MALLS). Each mutant was tested for tetramerization of the apo protein and with the addition of either GTP activator alone or GTP and additional dATP substrate. As with WT human SAMHD1, these experiments showed that no human SAMHD1 mutants tetramerize in the apo form or with the addition of GTP alone (*SI Appendix*, Fig. S10). On incubation with GTP and substrate dATP, all of the mutants underwent tetramerization with similar efficiency to WT, suggesting that they were not positively selected for this purpose (*SI Appendix*, Fig. S11). The degrees of phosphorylation of these variants were examined by Western blot using phospho-specific SAMHD1 antibodies (*SI Appendix*, Fig. S12). Variants Q594L and Q594R showed reduced signal relative to total SAMHD1 levels, suggesting that at least some positively selected sites do impinge on phosphorylation; however, differential binding affinity of the antibody for the mutated sites cannot be ruled out. Overall, introduction of these mutations did not seem to alter the protein expression levels greatly (*SI Appendix*, Fig. S12).

**Fig. 3.**
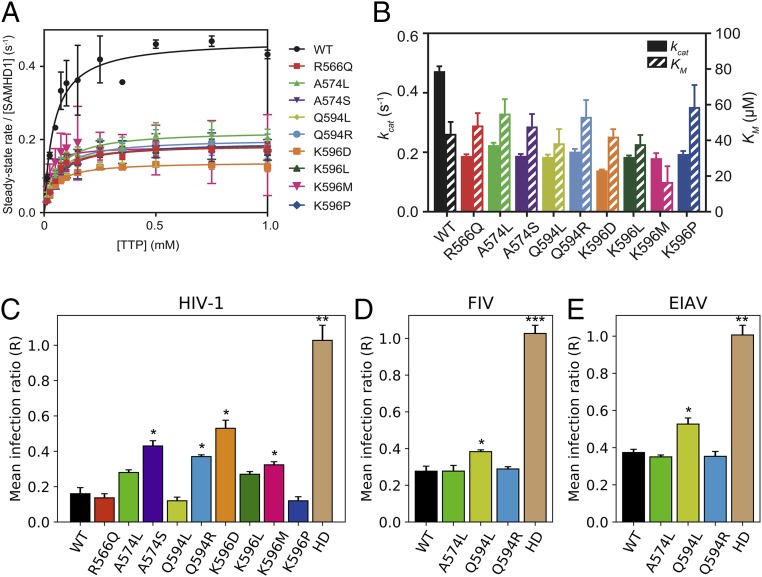
Activities of SAMHD1 positive selection point mutants. (*A*) Steady-state kinetics of triphosphohydrolase activity of human SAMHD1 WT and point mutants. Plots show the concentration dependence of the rate of SAMHD1 TTP hydrolysis in the presence of 0.2 mM GTP activator. The error bars are the SEM from triplicate measurements. (*B*) Kinetic parameters *k*_*cat*_ and *K*_*M*_ obtained from Michaelis–Menten analysis of data in *A*. Error bars are SEM from triplicate measurements. (*C*) Anti–HIV-1 restriction activity of human SAMHD1 WT and point mutants. The bars show the infection ratio of differentiated U937 cells expressing each variant with respect to an untransduced control. HD is the negative control human SAMHD1 active-site knockout mutant HD206/207AA. Error bars are the SEM from *n* = 3 or *n* = 4 measurements; asterisks indicate statistically significant difference from WT measured by Student’s *t* test and controlling false discovery rate by the Benjamini–Hochberg method. *P < 0.05; **P < 0.01; ***P < 0.001. (*D*) Anti-FIV restriction activity, as in *C*. (*E*) Anti-EIAV restriction activity, as in *C*.

We next assessed the triphosphohydrolase activities of the purified mutant enzymes. All SAMHD1 mutants had reduced (up to 2.5-fold) steady-state catalytic rates relative to WT, potentially due to a mismatch between the new residue and the human background sequence, but enzymatic activity was not severely disrupted (Fig. 3 *A* and *B*). The greatest effect was with mutant K596D, which, as mentioned above, marks the distinction between Old and New World primates (*SI Appendix*, Fig. S8). All mutants had similar *K*_*M*_ values to the WT, with mutations causing both increases and decreases in this parameter. One mutant, K596M, had a 3-fold reduction in *K*_*M*_, suggesting significantly enhanced substrate binding.

We then assessed the effect of changes at these sites under positive selection on the ability of SAMHD1 to restrict HIV-1 infection, measured in differentiated U937 cells using our previously described 2-color flow-cytometry HIV-1 restriction assay (23, 40). HIV-1 restriction was not ablated in any of the mutants tested, but appeared less efficient for several mutants, consistent with their observed reduction in enzymatic activity (Fig. 3*C*). Of note, a few variants (R566Q, Q594L, and Q596P) restricted HIV-1 infection as efficiently as WT, despite some reduction of enzymatic activity. We expanded our analysis to other lentiviruses, equine infectious anemia virus (EIAV) and FIV, comparing the effects of substitutions A574L (found in both horse and cat), Q594R (cat), Q594L (horse) (*SI Appendix*, Figs. S6 and S7), and WT human SAMHD1 on virus restriction (Fig. 3 *D* and *E*). Notably, variants found in cat conferred stronger restriction against FIV and EIAV than HIV-1, whereas the horse variant was relatively weaker, which paralleled their enzymatic activity, with Q594L being rather less active than A574L or Q594R (Fig. 3 *A* and *B*). Interestingly, the reverse was also true, as Q594L conferred strong restriction of HIV-1, but weaker EIAV/FIV restriction. Taken together, these results demonstrate that positively selected sites around T592 modulate enzyme activity, lentiviral restriction, and phosphorylation and provide insights into SAMHD1 regulation and evolution.

## Discussion

Viruses impose a significant burden upon their hosts, forcing them into persistent evolutionary conflict. Host antiviral responses must be carefully modulated, however, to prevent self-damage or dysregulation of pathways critical for cell homeostasis. There is evidence that such modulation can be achieved by controlling levels of transcription of innate immunity genes (41), but it has been unclear whether regulation of the activity of innate immunity proteins is also important. By investigating the deep evolutionary history of the innate immune protein SAMHD1, our results suggest that this mechanism might indeed be important and afford appropriate modulation.

To date, SAMHD1 evolution has been closely studied in primate lentiviruses and their hosts, where the characteristic signatures of positive diversifying selection have been observed, specifically at the N- and C-terminal regions that interact with Vpr/Vpx viral proteins (17, 19). It has remained unclear whether this coevolution has been restricted to primates and whether the activity of SAMHD1 might be modulated by the host in response to pathogens. We have found that positive selection in SAMHD1 is not restricted to primates, but rather has been pervasive throughout mammalian evolution and can be observed in every mammal group for which data are available (Fig. 1). This reflects either widespread similar adaptation to recent pathogens in a wide range of mammals or, perhaps more likely, an ancient and ongoing battle between the mammalian innate immune system and viruses, potentially involving other restriction factors and taxonomic groups.

We found that regions of SAMHD1 are under positive selection in multiple mammal groups, such as the C-terminal Vpx/Vpr binding site, which is under positive selection in Glires, Cetartiodactyla, Chiroptera, and Other Mammals (Fig. 1 and *SI Appendix*, Fig. S4). The surfaces of primate SAMHD1 bound by Vpx/Vpr appear to vary and even fluctuate throughout evolutionary time (18), but the density of sites identified in this region suggests that viral antagonists in other species may also target this region. The signatures of positive selection in mammalian SAMHD1 may represent both ongoing evolutionary conflict and a record of past battles with viruses now extinct.

Several classes of DNA-producing viruses are restricted in the presence of SAMHD1 (3–10), suggesting that many diverse animal viruses have an evolutionary incentive to overcome its effects. SAMHD1 sensitivity extends to FIV and EIAV, although there is no evidence of SAMHD1 antagonism by these or other lentiviruses, suggesting that, like HIV-1, their replication strategies do not require it (10). Nonetheless, ancient endogenous retrovirus sequences have been found in diverse species genomes (42), indicating that retroviruses have coevolved with animals and their restriction factors throughout metazoan evolution and may have imposed selective pressure on their hosts’ SAMHD1.

We identified a significant clustering of sites under positive selection in the domain containing the phosphorylation site T592, in mammals in general as well as in Cetartiodactyla, Chiroptera, and Other Mammals. Replacing human SAMHD1 residues at sites under positive selection in this domain with those found in other species—thereby mimicking possible residue substitutions due to positive selection—modestly enhanced tetramerization and substrate binding (*K*_*M*_), but reduced the catalytic rate (*k*_*cat*_) (*SI Appendix*, Fig. S11 and Fig. 3 *A* and *B*). In the case of the Q594L and Q594R variants, SAMHD1 phosphorylation also appeared to be reduced (*SI Appendix*, Fig. S12). Several of the mutations that decreased enzymatic activity also reduced restriction of HIV-1 (Fig. 3 *A*–*C*), demonstrating that sites distant from the active site and under positive selection have important influence on SAMHD1 function. Furthermore, we observed that substitutions normally found in cat and horse resulted in loss of anti–HIV-1 activity, but maintained restriction of FIV and EIAV, suggesting changes in SAMHD1 antiviral specificity over evolutionary time. These results suggest that positive selection in the regions we observed involves the modulation of SAMHD1’s activity, which might be differentially adapted in different species, perhaps due to the different biological properties of reverse transcriptase (RT) from these lentiviruses (43, 44). More generally, adaptation to each host’s range of pathogens may therefore have driven the widespread positive selection we observe.

There is an interesting parallel with the IFN-induced transmembrane 3 protein (IFITM3), which restricts diverse enveloped viruses. Several posttranslational modifications are determined by IFITM3’s N terminus, and replacing residues here with those found in nonhuman primate orthologs resulted in opposite effects on restriction of different viruses, similarly suggesting a trade-off in viral specificity (45). Meanwhile, SAMHD1 is in contrast with the rodent transferrin receptor (TfR1), which has undergone positive selection in regions bound by viruses to mediate cell entry: Mutations at these positions disrupt TfR1-dependent virus entry, but without influencing TfR1’s core functions involved with iron transport, suggesting effective separation between selection pressures to avoid pathogen interaction and retain biological activity (46).

One possible explanation is positive diversifying selection on SAMHD1’s function as a dNTP regulator, independent of its role as a restriction factor. However, it is not clear why an enzyme fulfilling an important homeostatic function would be under diversifying selection, and, moreover, there is no known precedent for diversifying selection to be associated with enzyme activity/regulation. Alternatively, substitutions driven by selection pressure from viruses might impact SAMHD1 enzymatic activity, resulting in selection for compensatory substitutions. The surface surrounding T592 may itself constitute an interaction interface, meaning that positive selection in this domain has occurred to disrupt interaction with viral proteins. However, the sites identified as under positive selection do not form an obvious binding surface, since some are buried. A third possibility is that SAMHD1 regulation is directly involved in the virus–host evolutionary conflict, adapting catalytic efficiency or propensity for (de)phosphorylation to regulate antiviral potency. For instance, sites 618 and 619, which were identified as under positive selection in mammals, nonprimates, and individually in Carnivora (site 618) and Chiroptera (site 619), have been implicated in binding with cyclin A2, an interaction responsible for the phosphorylation of SAMHD1 T592 (28).

In this study, we have found evidence of an arms race between viruses and their hosts of a significantly larger scale than previously realized. Far from being limited to primates, we have found that positive selection has occurred throughout the evolution of SAMHD1 in mammals, most likely due to selection pressure applied by modern or extinct DNA-producing viruses. Significantly, we have found evidence that this adaptation can involve modulation of a host protein’s function. This suggests a model in which SAMHD1 is under selective constraints to both conserve its metabolic and antiviral functions while continually adapting to pressures imposed by viruses. This principle of balancing function conservation with continual adaptation is likely to apply to other innate immunity components, as they adapt to manage the cost of evolutionary conflict.

## Methods

### Licensing for Human or Animal Materials.

This work involved no experiments using human or animal materials.

### Sequence Data and Alignment.

Mammalian SAMHD1 DNA sequences were collected by using NCBI BLAST (blastn algorithm) with human SAMHD1 coding sequence (accession no. NM_015474.3) as the query. Mostly, these were predicted mRNA sequences originating from automated analysis of mammal genome sequences, while the majority of primate sequences originated from previous SAMHD1 studies (17, 19). Where more than 1 sequence was available from a single species (usually predicted transcript variants), sequences most closely matching the human sequence were selected. The sequence for Tasmanian devil (*Sarcophilus harrisii*) was found to be divided into 2 sequence records (accession nos. XM_003758997.2 and XM_012553363.1); these were concatenated to give a full-length sequence. Preliminary phylogenetic analysis including nonmammal taxa found the platypus (*Ornithorhynchus anatinus*) sequence incorrectly positioned outside of the mammalian clade; this was therefore excluded from subsequent analysis. The list of species and accession numbers for sequences used are listed in *SI Appendix*, Table S1. Sequences that were <70% of the length of the human SAMHD1 sequence were excluded.

The nucleotide coding sequences were initially aligned as translated protein by using MUSCLE [Version 3.8.31; ref. 47, as implemented in SEAVIEW (Version 4.4.0); ref. 48] and then further edited manually, with a highly conservative approach: Sections within sequences which could not be aligned with high confidence were masked, such that they would be treated as missing data (equivalent to alignment gaps) by phylogenetics tools. Alignment columns containing no data (exclusively gaps or masked codons) in 20% of sequences were removed.

### Phylogeny Estimation.

A phylogenetic tree was estimated by maximum likelihood using RAxML (Version 7.7.2; refs. 49 and 50) with the general time-reversible substitution model and gamma-distributed rate heterogeneity. Confidence in the tree topology was assessed by estimating trees from 1,000 nonparametric bootstrap samples (51). In repeating selection analyses with alternative tree topologies, we used 3 of the nonparametric bootstrap trees generated by RAxML that had independent maximum parsimony trees as initial estimates. Tree figures were produced by using FigTree (Version 1.3.1) (52).

### Selection Analysis.

Selection analyses were performed by using the codeml program of the PAML package (Version 4.7a; ref. 34). We used the site-specific tests of positive selection M1a/M2a (32) and M7/M8 (33). For the large all-mammals dataset, the more complex M8 model was unable to converge, and for mammal subgroups, sites identified with M2a were almost always subsets of those identified with M8.

To reduce the risk of the log-likelihood optimization reaching a local optimum, all program runs were performed 5 times with different initial parameters for the transition/transversion ratio (κ) and *dN/dS* ratio (ω): 0.1, 1, and 10. Tree branch lengths were first optimized with codeml’s model 0 (which allows a single ω value) with the corresponding initial parameter values, and these branch lengths were used as starting values in subsequent analyses with more complex models. Codon stationary frequencies were estimated by using the F1x4 model by default, but F3x4 was used if optimization difficulties were encountered with F1x4 (null and alternative models were always compared by using consistent codon frequency models; *SI Appendix*, Table S2).

Statistical justification for the alternative model was assessed by using the likelihood-ratio test, using 2 degrees of freedom. Sites were identified as being under positive selection if the computed Bayes empirical Bayes probability for the site belonging to the positive selection class was >0.95.

### Clustering of Sites under Positive Selection.

Statistical significance for sites under positive selection clustering in regions of the SAMHD1 linear sequence was determined by combinatorial analysis. Briefly, in a sequence of *N* = 626 sites, of which *n* are under positive selection, there is a region of biological interest comprising *R* sites (*R* < *N*), of which *r* are under positive selection (*r* ≤ *n*). The probability of *r* or more of the *n* positively selected sites occurring in this region at random is given by

$$\frac{\sum_{s^{'}\geq r}\left(\frac{R}{s^{'}}\right)\left(\frac{N-R}{n-s^{'}}\right)}{\left(\begin{array}{c} N\\ n \end{array}\right)}.$$

### Protein Expression and Purification.

The DNA sequence for human SAMHD1 (residues M1–M626) was amplified by PCR from plasmid template and inserted into a pET52b expression vector (Novagen) using ligation independent cloning to produce an N-terminal StrepII-tag fusion protein. Point mutations corresponding to the residues found in other species were introduced into the WT protein construct by using the Quikchange II kit. All insert sequences were verified by DNA sequencing. Strep-tagged SAMHD1 constructs were expressed in the *E. coli* strain Rosetta 2 (DE3) grown at 37 °C with shaking. Protein expression was induced by addition of 0.1 mM isopropyl β-d-thiogalactopyranoside (IPTG) to log-phase cultures (*A*_600_ = 0.5), and the cells were incubated for a further 20 h at 18 °C. Cells were harvested by centrifugation resuspended in 30 mL of lysis buffer [50 mM Tris⋅HCl pH 7.8, 500 mM NaCl, 4 mM MgCl_2_, 0.5 mM Tris(2-carboxyethyl)phosphine (TCEP), 1× EDTA-free mini complete protease inhibitors (Roche), and 0.1 U/mL Benzonase (Novagen)] per pellet of 1 L of bacterial culture and lysed by disruption in EmulsiFlex-C5 homogenizer (Avestin). The lysate was cleared by centrifugation for 1 h at 48,000 × *g* and 4 °C, then applied to a 10-mL StrepTactin affinity column (IBA) followed by 600 mL of wash buffer (50 mM Tris⋅HCl, pH 7.8, 500 mM NaCl, 4 mM MgCl_2_, and 0.5 mM TCEP) at 4 °C. Bound proteins were eluted from the column by circulation of 1 mg of 3C protease (GE) in 10 mL of wash buffer over the column in a closed circuit overnight. The 3C protease was removed by incubation of the eluent with 500 µL of GSH-Sepharose (GE). After centrifugation to remove the resin, the supernatant was concentrated to 5 mL and applied to a Superdex 200 16/60 (GE) size-exclusion column equilibrated with 10 mM Tris⋅HCl (pH 7.8), 150 mM NaCl, 4 mM MgCl_2_, and 0.5 mM TCEP. Peak fractions were concentrated to ∼20 mg/mL and flash-frozen in liquid nitrogen in small aliquots.

### SEC-MALLS.

SEC-MALLS was used to determine the molar mass composition of SAMHD1 samples upon addition of deoxynucleotide/nucleotide substrates (dATP; 500 µM) and activators (GTP; 200 µM). Samples (30 µM SAMHD1 and variants) were incubated with substrate and activator at 4 °C for 5 min, and then 100 µL was applied to a Superdex 200 10/300 GL column equilibrated in 20 mM Tris⋅HCl, 150 mM NaCl, 5 mM MgCl_2_, 0.5 mM TCEP, and 3 mM NaN_3_ (pH 8.0) at a flow rate of 0.5 mL/min. The scattered light intensity and protein concentration of the column eluate were recorded by using a DAWN-HELEOS laser photometer and an OPTILAB-rEX differential refractometer (dRI) (d*n*/d*c* = 0.186), respectively. The weight-averaged molecular mass of material contained in chromatographic peaks and peak integrals were determined by using the combined data from both detectors in the ASTRA software (Version 6.1; Wyatt Technology Corp.).

### SAMHD1 Catalytic Activity Assays.

The hydrolysis of dNTPs by SAMHD1 and SAMHD1 variants was quantified by using a coupled assay utilizing the biosensor MDCC-PBP (53, 54) to measure phosphate release from combined SAMHD1 triphosphohydrolase and *Saccharomyces cerevisiae* Ppx1 exopolyphosphatase activity as described (55). In a typical experiment, solutions containing SAMHD1 constructs, Ppx1, MDCC-PBP, and GTP were incubated for 5 min in assay buffer (20 mM Tris, pH 8.0, 150 mM NaCl, 5 mM MgCl_2_, and 2 mM TCEP) at 25 °C before the reaction was initiated by the addition of substrate [thymidine triphosphate (TTP)]. The final concentrations were 100 nM SAMHD1, 10 nM Ppx1, 40 µM MDCC-PBP, 0.2 mM GTP, and varying concentration of TTP. The fluorescence intensity was recorded at 430-nm excitation and 465-nm emission over a period of 10–30 min in a Clariostar multiwall plate reader (BMG). Steady-state rates were obtained from time courses of P_i_ formation by linear regression of the data points in the linear phase of the reaction. Rates were divided by the SAMHD1 concentration and plotted versus substrate concentration. Apparent dissociation constants for substrate binding (*K*_*M*_) and catalytic constants (*k*_*cat*_) were then determined by nonlinear least-squares fitting using either a hyperbolic or a Hill function. All measurements were performed in duplicate or triplicate.

### SAMHD1 Plasmids.

WT SAMHD1 was previously cloned into the pLGatewayIeYFP vector to create pLGateway_SAMHD1IRESYFP (23). SAMHD1 mutants (R566Q, A574S, A574L, Q594L, Q594R, K596D, K596P, K596L, and K596M) were created by PCR-based (Pfu; Agilent) site-directed mutagenesis of the pLGateway_SAMHD1IRESYFP vector using the manufacturer’s protocol and the primers listed in *SI Appendix*, Table S8.

### Cells and Virus Production.

The 293T cells were maintained in Dulbecco’s modified Eagle medium (Life Technologies) and U937 cells in RPMI +[l]- glutamine (Life Technologies), each supplemented with 10% heat-inactivated fetal bovine serum (Labtech) and 1% penicillin/streptomycin (Sigma). Moloney murine leukemia virus-based SAMHD1 and yellow fluorescent protein (YFP)-expressing virus-like particles (VLPs) were produced by cotransfecting 293T cells with pVSV-G (56), pKB4 (57), and pLGateway_SAMHD1IRESYFP (WT or mutants) into 293T cells and harvesting 48 h after transfection. HIV-1-GFP was made by cotransfection of pVSV-G, p8.91 (58), and pCSGWGFP (59). EIAV-GFP vector was made with plasmids pEIAV-SIN6.1 CGFPW, pEV53D (gift of John Olsen, Addgene, Chapel Hill, NC; ref. 60), and pVSV-G. FIV-GFP vector was made by using plasmids pGIN SIN FIVGFP, pFP93 (gift of Eric Poeschla, University of Colorado, Denver), and pVSV-G (61). Plasmids were mixed at ratio 3:3:3 μg/10-cm dish. Supernatants were collected 48 and 72 h posttransfection, centrifuged at 3,000 rpm, filtered through a 0.45-μm filter, and concentrated on the same day by centrifugation at 100,000 × *g* for 2 h at 4 °C through a 20% sucrose cushion before resuspension in serum-free RPMI. VLPs were titered on U937 cells for normalization prior to infection. Viral stocks were stored at −80 °C until use.

### HIV-1 Restriction Assay.

Undifferentiated U937 cells (3 × 10^5^ cells in 12-well plate) were transduced with SAMHD1-YFP VLPs by spinoculation at 800 × *g* for 90 min in the presence of 10 µg/mL polybrene. After 72 h incubation, the cells were passaged 1:4 and differentiated with 100 nM phorbol 12-myristate 13-acetate (Sigma) for 96 h. Differentiated cells were infected in triplicate with HIV-1-GFP in the presence of 10 µg/mL polybrene, and restriction was assessed after 72 h by 2-color flow cytometry using a Fortessa ×20 analyzer (BD Biosciences). Data were analyzed by using the FlowJo software suite. Restriction was calculated by dividing the percentage of SAMHD1-expressing (YFP +ve) cells that were infected with HIV-1 (GFP +ve) by the percentage of SAMHD1-negative cells that were infected to give an infection ratio, *R*.

### Immunoblotting.

As described above, SAMHD1-YFP–expressing U937 cells were sorted on MoFlo XDP (Beckman Coulter). After culturing for 3–4 d to recover from cell sorting, 1 × 10^7^ cells were harvested and washed twice with phosphate-buffered saline. Supernatant was removed, and cells were lysed with 100 μg of radioimmunoprecipitation assay buffer (ThermoFisher) in the presence of protease inhibitors (Roche), DNase (Invitrogen), and phosphatase inhibitor (ThermoFisher) at 4 °C for 1 h. The lysates were subsequently sonicated for 5 min, ×2 pulses, and 40% amplitude and centrifuged for 30 min at 4 °C and 48,000 × *g*. Laemmli buffer (2×, Sigma) was added at a 1:1 ratio to the lysate and boiled at 95 °C for 5 min. To separate proteins, samples were loaded on sodium dodecyl sulfate-polyacrylamide gel electrophoresis and transferred onto polyvinylidene fluoride membranes (Millipore), which were then incubated with blocking buffer (5% bovine serum albumin and 0.05% Tween 20 in Tris⋅HCl buffer, pH 7.5; Sigma) at 4 °C overnight. Mouse anti-SAMHD1 1F6 monoclonal antibody (GeneTex; 1:500) and rabbit anti-phosphorylated-SAMHD1 monoclonal antibody (CST; 1:1,000) were used as primary antibodies. Corresponding secondary antibodies used were goat anti-mouse IRDye800CW (LI-COR; 1:10,000) and goat anti-rabbit IRDye800CW (LI-COR; 1:10,000). Fluorescence was measured by using the LI-COR Odyssey imaging (LI-COR Bioscience).

## Supplementary Material

Supplementary File
