# Supplementary material for: Positive selection in dNTPase SAMHD1 throughout mammalian evolution
Source: Proc Natl Acad Sci U S A. 2019 Aug 26;116(37):18647–54. doi: 10.1073/pnas.1908755116 (PMC6744909; doi:10.1073/pnas.1908755116)
Supplement: Supplementary File [file pnas.1908755116.sapp.pdf]

PNAS
www.pnas.org

Supplementary Information for

Positive Selection in dNTPase SAMHD1 Throughout Mammalian Evolution

Christopher Monit[a], Elizabeth R. Morris[b], Christopher Ruis[a], Bart Szafran[c], Grant Thiltgen[a], Chloe Ming-Han Tsai[d], N. Avrion Mitchison[a], Kate N. Bishop[d], Jonathan P. Stoye[c], Ian A. Taylor[b], Ariberto Fassati[a,*], and Richard A. Goldstein[a,*]

Ariberto Fassati
Email: a.fassati@ucl.ac.uk

Richard A. Goldstein
Email: r.goldstein@ucl.ac.uk,

This PDF file includes:

Supplementary text
Tables S1 to S8
Figures S1 to S12
SI References

www.pnas.org/cgi/doi/10.1073/pnas.1908755116

Supplementary Table S1: Species represented in SAMHD1 analyses, with GenBank accession numbers for the DNA sequences used.

Analysis group	Common name	Taxonomic name	Sequence accession
Carnivora	amur tiger	*Panthera tigris altaica*	XM_007077693.1
Carnivora	cat	*Felis catus*	XM_003983547.2
Carnivora	dog	*Canis lupus familiaris*	XM_542986.4
Carnivora	ferret	*Mustela putorius furo*	XM_004746473.1
Carnivora	giant panda	*Ailuropoda melanoleuca*	XM_002915170.2
Carnivora	polar bear	*Ursus maritimus*	XM_008698122.1
Carnivora	walrus	*Odobenus rosmarus divergens*	XM_004393158.1
Carnivora	weddell seal	*Leptonychotes weddellii*	XM_006733646.1
Cetartiodactyla	alpaca	*Vicugna pacos*	XM_006202728.1
Cetartiodactyla	bactrian camel	*Camelus bactrianus*	XM_010960760.1
Cetartiodactyla	baiji	*Lipotes vexillifer*	XM_007447732.1
Cetartiodactyla	common bottlenose dolphin	*Tursiops truncatus*	XM_004326928.1
Cetartiodactyla	cow	*Bos taurus*	NM_001075861.1
Cetartiodactyla	dromedary camel	*Camelus dromedarius*	XM_010975213.1
Cetartiodactyla	goat	*Capra hircus*	XM_013968902.1
Cetartiodactyla	minke whale	*Balaenoptera acutorostrata scammoni*	XM_007193161.1
Cetartiodactyla	mouflon or sheep	*Ovis aries musimon*	XM_012114778.1
Cetartiodactyla	orca	*Orcinus orca*	XM_004272978.2
Cetartiodactyla	plains or american bison	*Bison bison bison*	XM_010829204.1
Cetartiodactyla	sheep	*Ovis aries*	XM_012189160.1
Cetartiodactyla	sperm whale	*Physeter catodon*	XM_007116777.1
Cetartiodactyla	tibetan antelope	*Pantholops hodgsonii*	XM_005968060.1
Cetartiodactyla	water buffalo	*Bubalus bubalis*	XM_006051717.1
Cetartiodactyla	wild bactrian camel	*Camelus ferus*	XM_006188650.1
Cetartiodactyla	wild boar	*Sus scrofa*	NM_001292105.1
Cetartiodactyla	yak	*Bos mutus*	XM_005896009.1
Chiroptera	big brown bat	*Eptesicus fuscus*	XM_008149758.1
Chiroptera	black flying fox	*Pteropus alecto*	XM_006921792.1
Chiroptera	brandts bat	*Myotis brandtii*	XM_005868463.1
Chiroptera	davids myotis bat	*Myotis davidii*	XM_006764508.1
Chiroptera	large flying fox	*Pteropus vampyrus*	XM_011366586.1
Chiroptera	little brown bat	*Myotis lucifugus*	XM_006089575.1
Glires	brown rat	*Rattus norvegicus*	NM_001191743.1
Glires	chinese hamster	*Cricetulus griseus*	XM_007625631.1
Glires	damara mole rat	*Fukomys damarensis*	XM_010640022.1
Glires	deer mouse	*Peromyscus maniculatus bairdii*	XM_006984164.1
Glires	degu	*Octodon degus*	XM_004630995.1
Glires	european rabbit	*Oryctolagus cuniculus*	XM_008256128.1
Glires	golden hamster	*Mesocricetus auratus*	XM_005084653.1
Glires	guinea pig	*Cavia porcellus*	XM_003467744.2
Glires	house mouse	*Mus musculus*	NM_018851.3
Glires	lesser egyptian jerboa	*Jaculus jaculus*	XM_004666629.1
Glires	long tailed chinchilla	*Chinchilla lanigera*	XM_005384981.1
Glires	naked mole rat	*Heterocephalus glaber*	XM_004888567.1
Glires	pika	*Ochotona princeps*	XM_004586040.1
Glires	prairie vole	*Microtus ochrogaster*	XM_005363152.1
Glires	thirteen lined ground squirrel	*Spermophilus tridecemlineatus*	XM_005329859.1
Glires	upper galilee mountains mole rat	*Nannospalax galili*	XM_008836546.1
Primates	agile gibbon	*Hylobates agilis*	JQ231127.1
Primates	allens swamp monkey	*Allenopithecus nigroviridis*	JN936900.1
Primates	angola colobus	*Colobus angolensis palliatus*	JN936905.1
Primates	angolan talapoin	*Miopithecus talapoin*	JN936901.1
Primates	black capped squirrel monkey	*Saimiri boliviensis boliviensis*	XM_010342629.1
Primates	black white ruffed lemur	*Varecia variegata variegata*	JN936913.1
Primates	bonobo	*Pan paniscus*	NM_001279186.1
Primates	bornean orangutan	*Pongo pygmaeus*	JN936888.1
Primates	brown woolly monkey	*Lagothrix lagotricha*	JQ231150.1
Primates	chimp	*Pan troglodytes*	NM_001280510.1
Primates	collared mangabey	*Cercocebus torquatus*	JQ231133.1
Primates	colobus	*Colobus guereza*	JQ231145.1
Primates	common marmoset	*Callithrix jacchus*	JN936906.1
Primates	common squirrel monkey	*Saimiri sciureus*	JN936909.1
Primates	cotton tamarin	*Saguinus oedipus*	JN936908.1
Primates	crab eating macaque	*Macaca fascicularis*	NM_001287721.1
Primates	de brazzas monkey	*Cercopithecus neglectus*	JQ231141.1
Primates	diana monkey	*Cercopithecus diana*	JN936902.1

Primates	drill	*Mandrillus leucophaeus*	JQ231131.1
Primates	francois langur	*Trachypithecus francoisi*	JN936904.1
Primates	gelada	*Theropithecus gelada*	JN936896.1
Primates	geoffroys spider monkey	*Ateles geoffroyi*	JN936911.1
Primates	golden bellied mangabey	*Cercocebus chrysogaster*	JN936898.1
Primates	golden snub nosed monkey	*Rhinopithecus roxellana*	XM_010355318.1
Primates	gorilla	*Gorilla gorilla*	NM_001279619.1
Primates	gray mouse lemur	*Microcebus murinus*	JN936914.1
Primates	green monkey	*Chlorocebus sabaeus*	NM_001292080.1
Primates	grivet	*Chlorocebus aethiops*	KF741041.1
Primates	hamadryas baboon	*Papio hamadryas*	JN936890.1
Primates	human	*Homo sapiens*	NM_015474.3
Primates	lar gibbon	*Hylobates lar*	JN936889.1
Primates	mandrill	*Mandrillus sphinx*	JN936897.1
Primates	mantled howler	*Alouatta palliata*	JN936912.1
Primates	nancy mas night monkey	*Aotus nancymaae*	XM_012455058.1
Primates	northen greater galago	*Otolemur garnettii*	XM_003788255.1
Primates	olive baboon	*Papio anubis*	NM_001279525.1
Primates	patas monkey	*Erythrocebus patas*	JQ231138.1
Primates	philippine tarsier	*Tarsius syrichta*	XM_008049665.1
Primates	proboscis monkey	*Nasalis larvatus*	JQ231144.1
Primates	pygmy marmoset	*Callithrix pygmaea*	JQ231146.1
Primates	red bellied titi	*Callicebus moloch*	JQ231152.1
Primates	red shanked douc	*Pygathrix nemaeus nemaeus*	JN936903.1
Primates	rhesus macaque	*Macaca mulatta*	JQ231135.1
Primates	siamang	*Symphalangus syndactylus*	JQ231128.1
Primates	sooty mangabey	*Cercocebus atys*	JQ231132.1
Primates	southern pig tailed macaque	*Macaca nemestrina*	XM_011767026.1
Primates	sumatran orangutan	*Pongo abelii*	XM_002830274.3
Primates	tantalus monkey	*Chlorocebus tantalus*	JN936892.1
Primates	three striped night monkey	*Aotus trivirgatus*	JN936907.1
Primates	tufted capuchin	*Cebus apella*	JN936910.1
Primates	vervet monkey	*Chlorocebus pygerythrus*	JQ231137.1
Primates	white cheeked gibbon	*Nomascus leucogenys*	NM_001280119.1
Primates	white faced saki	*Pithecia pithecia*	JQ231151.1
Primates	white lipped tamarin	*Saguinus labiatus*	JQ231147.1
Primates	wolfs mona monkey	*Cercopithecus wolfi*	JQ231140.1
Other	aardvark	*Orycteropus afer afer*	XM_007934742.1
Other	african bush elephant	*Loxodonta africana*	XM_003411700.2
Other	armadillo	*Dasypus novemcinctus*	XM_004466604.2
Other	cape elephant shrew	*Elephantulus edwardii*	XM_006881709.1
Other	cape golden mole	*Chrysochloris asiatica*	XM_006875733.1
Other	common shrew	*Sorex araneus*	XM_004612642.1
Other	european hedgehog	*Erinaceus europaeus*	XM_007518471.1
Other	gray short tailed opposum	*Monodelphis domestica*	XM_001381548.3
Other	horse	*Equus caballus*	XM_001499498.3
Other	lesser hedgehog tenrec	*Echinops telfairi*	XM_004698253.1
Other	przewalskis horse	*Equus przewalskii*	XM_008541885.1
Other	star nosed mole	*Condylura cristata*	XM_004686953.1
Other	sunda flying lemur	*Galeopterus variegatus*	XM_008575366.1
Other	tasmanian devil (5')	*Sarcophilus harrisii*	XM_003758997.2
Other	tasmanian devil (3')	*Sarcophilus harrisii*	XM_012553363.1
Other	treeshrew	*Tupaia chinensis*	XM_006144103.1
Other	west indian manatee	*Trichechus manatus latirostris*	XM_004370368.1
Other	white rhinoceros	*Ceratotherium simum simum*	XM_004430428.1

Supplementary Table S2: Log-likelihood values, test statistics and *p* values for analyses of mammalian SAMHD1. Each model fitting was done with 5 different initial parameter values (see methods) and the best fitting models are shown. Abbreviations: Top., tree topology; ML, maximum likelihood tree topology; T_i, alternative topology; Null/Alt., null/alternative model; lnL, log-likelihood; *D*, test statistic for likelihood ratio test; d.f., degrees of freedom for chi square distribution (the difference in number of free parameters between null and alternative models); Freq., model of codon frequency used (see methods); BS, branch-site specific model with $\omega \geq 1$ on foreground branches; BS (ω =1), branch-site model with ω fixed at 1 on foreground branches; f.g., branches foreground.

Dataset	Top.	Null	Alt.	Null lnL	Alt. lnL	*D*	d.f.	*p* value	Freq.
All mammals	ML	M1a	M2a	-39160.68	-38954.72	411.93	2	3.55E-90	F1X4
Mammals exc. primates	ML	M1a	M2a	-33185.82	-33034.90	301.84	2	2.86E-66	F1X4
All mammals	T_1	M1a	M2a	-39239.19	-39029.14	420.10	2	5.98E-92	F1X4
All mammals	T_2	M1a	M2a	-39233.30	-39035.09	396.42	2	8.29E-87	F1X4
All mammals	T_3	M1a	M2a	-39176.35	-38971.45	409.80	2	1.03E-89	F1X4
Carnivora	ML	M1a	M2a	-4683.41	-4672.52	21.78	2	1.86E-05	F3X4
Chiroptera	ML	M1a	M2a	-4733.32	-4716.64	33.36	2	5.70E-08	F1X4
Glires	ML	M1a	M2a	-12913.41	-12901.97	22.88	2	1.08E-05	F1X4
Primates	ML	M1a	M2a	-8298.97	-8282.41	33.12	2	6.42E-08	F1X4
Cetartiodactyla	ML	M1a	M2a	-5957.73	-5928.79	57.87	2	2.72E-13	F1X4
Other Mammals	ML	M1a	M2a	-13740.77	-13723.18	35.17	2	2.31E-08	F1X4
Carnivora	ML	M7	M8	-4684.21	-4672.55	23.32	2	8.62E-06	F3X4
Chiroptera	ML	M7	M8	-4704.80	-4666.78	76.02	2	3.10E-17	F3X4
Glires	ML	M7	M8	-12902.26	-12884.58	35.36	2	2.09E-08	F1X4
Primates	ML	M7	M8	-8280.43	-8242.52	75.83	2	3.42E-17	F3X4
Cetartiodactyla	ML	M7	M8	-5936.35	-5890.21	92.27	2	9.19E-21	F3X4
Other Mammals	ML	M7	M8	-13672.56	-13613.78	117.57	2	2.94E-26	F1X4

Supplementary Table S3: Sites identified in mammalian SAMHD1, using codeml site models M2a or M8. '+' indicates Bayes empirical Bayes probability >0.95. Abbreviations: Res., human SAMHD1 residue; Ma., all mammals (M2a); No Pr., all mammals without primates (M2a); Ca., Carnivora (M8); Ch., Chiroptera (M8); Gl., Glires (M8); Pr., Primates (M8); Ce., Cetartiodactyla (M8); O.M., Other Mammals (M8); Lag., sites from Laguette *et al.,* ref (1); Lim, sites from Lim *et al.,* ref. (2).

Site	Res.	Ma.	No Pr.	Ca.	Ch.	Gl.	Pr.	Ce.	O. M.	Lag.	Lim
3	R				+						
15	C									+	
32	W	+	+							+	+
36	L									+	+
46	G										+
55	R	+	+		+						
56	R		+			+					
57	G	+	+								
60	E	+	+		+	+					
63	V	+								+	
68	I				+						
69	R										+
79	P				+						
107	L										+
112	V	+								+	
114	T				+						
115	M				+						
256	Q	+			+		+			+	
266	C				+						
280	V	+	+						+		
282	D								+		
283	S	+			+		+				
284	L	+	+			+			+		
346	E				+						
395	D									+	
408	R	+					+			+	
442	R						+			+	
463	T	+	+		+			+		+	
464	G							+			
465	Q	+	+								
466	I	+	+					+		+	
469	K		+		+						
472	D	+	+		+						
473	Y				+						
475	S	+	+	+				+	+		
482	S			+							
486	K	+									+

**488**	**L**	+	+		+	+			+	+	
**563**	**Y**				+						
**566**	**R**	+	+								
**574**	**A**	+	+		+						
**575**	**D**	+	+						+		
**586**	**V**							+			
**594**	**Q**	+	+					+			
**596**	**K**	+	+		+			+	+		
**600**	**D**	+	+					+			
**601**	**S**	+	+	+						+	
**602**	**T**									+	
**604**	**V**							+			
**605**	**Q**					+					
**609**	**R**	+									
**610**	**L**	+	+			+					
**611**	**R**	+	+								
**614**	**S**	+	+		+			+	+	+	
**616**	**S**	+	+		+			+	+		
**618**	**V**	+	+	+				+		+	
**619**	**Q**	+	+		+						
**622**	**K**	+	+		+			+			
**623**	**D**				+			+			
**624**	**D**	+	+		+						
**625**	**P**	+	+			+					
**626**	**M**	+	+		+	+	+	+		+	
**Site**	**Res.**	**Ma.**	**No Pr.**	**Ca.**	**Ch.**	**Gl.**	**Pr.**	**Ce.**	**O. M.**	**Lag.**	**Lim**

Supplementary Table S4: Comparison of sites found to be under positive selection between maximum likelihood (ML) tree topology and three alternative topologies, derived from non-parametric bootstrap sampling of the same mammalian SAMHD1 sequence alignment. Columns show sites found in ML tree analysis only (not found with alternative topology), found with both (intersection) or with alternative topology only (not found with ML topology), respectively. Where there are discrepancies, the relevant sites are given in parentheses (human sequence numbering).

**Topology**	**T_i total**	**ML only**	**Intersection**	**T_i only**
T_1	38	None	36 sites	2 sites (56, 464)
T_2	36	3 (112, 594, 609)	33 sites	3 sites (36, 56, 602)
T_3	37	None	36 sites	1 site (36)

Supplementary Table S5: Probabilities of the number the sites identified as being under positive selection in regions of interest if their distribution throughout the SAMHD1 sequence were random.

SAMHD1 dataset	N-term. Vpx	T592 domain	C-term. Vpx
Mammals	0.3634	0.0048	0.0000
Mammals exc. Primates	0.2487	0.0011	0.0000
Carnivora	1.0000	0.1155	0.1279
Chiroptera	0.3194	0.0349	0.0000
Glires	0.2172	0.7421	0.0016
Primates	1.0000	1.0000	0.1573
Cetartiodactyla	1.0000	0.0039	0.0000
Other Mammals	1.0000	0.0367	0.0335

Supplementary Table S6: Investigation of predicted SAMHD1 structural homology. Model structures were estimated using SwissModel (3) for representative species in our dataset, using as a template a single chain taken from a tetrameric human SAMHD1 crystal structure (PDB 4TNP, chain D, the longest chain in this tetramer structure; (4)) and omitting the N- and C- terminal regions of the sequences which are not present in the template (residues 1-112 and 600-626). For comparisons involving a chain from the tetrameric mouse structure 6BRG (5), we likewise omitted large regions that were not present in both structures. Chicken SAMHD1 was also modelled as a more distant example. We measured the similarity between the human 4TNP-D structure and these structure models by solving for the minimum root mean square deviation (RMSD) between corresponding Cα atoms and also calculating the Z-score with respect to random structure alignments, both using the DALI webserver (6). To confirm that using 4TNP-D as a template did not exaggerate the similarity, we generated 20 'scrambled' random permutations of the 4TNP-D residues and estimated structure models for these sequences by the same approach, again using 4TNP-D as a template. Abbreviations: Min. RMSD, minimum root-mean square deviation; Number Align., the number of aligned residues between the model and structure; Num. res., number of residues present in the model; %id, per cent sequence identity.

[See next page]

SAMHD1	Template	Comparison	Z-score	Min. RMSD (Å)	Num. Align.	Num. res.	%id
mouse	4TNP-D	6BRG-A	49.4	1	419	491	99
mouse	4TNP-D	4TNP-D	57.2	0.3	477	491	80
human	4TNP-D	4TNP-D	60.1	0.2	482	482	100
sheep	4TNP-D	4TNP-D	59.6	0.2	481	486	84
cat	4TNP-D	4TNP-D	59.5	0.3	482	486	83
horse	4TNP-D	4TNP-D	59.9	0.1	482	486	87
brandts bat	4TNP-D	4TNP-D	64.0	0.3	482	486	79
opossum	4TNP-D	4TNP-D	64.5	0.2	482	486	78
chicken	4TNP-D	4TNP-D	63.4	0.2	483	487	70
scrambled 1	4TNP-D	4TNP-D	41.2	1.2	404	471	22
scrambled 2	4TNP-D	4TNP-D	34.5	1.2	386	432	21
scrambled 3	4TNP-D	4TNP-D	34.6	1.4	345	392	21
scrambled 4	4TNP-D	4TNP-D	33.2	1.4	309	335	23
scrambled 5	4TNP-D	4TNP-D	33.5	1.4	384	460	22
scrambled 6	4TNP-D	4TNP-D	31.5	1.4	366	397	20
scrambled 7	4TNP-D	4TNP-D	32.5	1.4	398	457	22
scrambled 8	4TNP-D	4TNP-D	30.7	1.4	301	339	21
scrambled 9	4TNP-D	4TNP-D	34.4	1.5	360	417	23
scrambled 10	4TNP-D	4TNP-D	26.8	1.5	333	384	22
scrambled 11	4TNP-D	4TNP-D	31.2	1.5	351	381	20
scrambled 12	4TNP-D	4TNP-D	26.6	1.5	296	338	20
scrambled 13	4TNP-D	4TNP-D	30.1	1.5	289	308	19
scrambled 14	4TNP-D	4TNP-D	20.4	1.5	266	300	23
scrambled 15	4TNP-D	4TNP-D	34.0	1.6	390	446	21
scrambled 16	4TNP-D	4TNP-D	33.9	1.6	383	444	21
scrambled 17	4TNP-D	4TNP-D	23.1	1.6	290	324	23
scrambled 18	4TNP-D	4TNP-D	30.2	1.7	340	410	22
scrambled 19	4TNP-D	4TNP-D	22.3	1.7	309	401	23
scrambled 20	4TNP-D	4TNP-D	24.7	1.7	314	352	22

Supplementary Table S7: Comparison of side chain position and orientation between mutant residues in human SAMHD1 background and non-human SAMHD1 proteins for whom these are wildtype (WT). We selected four sites under positive selection and one or more candidate mutant residues based on those observed in different mammal species. We then selected representative species for which each mutant residue is WT and from these mammal sequences estimated protein structure models using SwissModel (see **Supplementary Table S6**). Separately, we predicted structures for each individual mutant human SAMHD1 sequence by the same approach. We then aligned each of the mammal protein models to the corresponding human mutant model by minimising the root mean square deviation (RMSD) between homologous Cα atoms, excluding the residue of interest. With the resulting translation and rotation matrix, we then aligned the atoms for the individual residues of interest in the mammal and mutant structure and calculated the RMSD for these residue pairs alone.

SAMHD1 sequence	Mutation	Residue RMSD (Å)
squirrel monkey	R566Q	0.0074
Philippine tarsier	A574L	0.0539
squirrel monkey	A574S	0.0195
white rhinoceros	Q594L	0.0859
sheep	Q594R	0.7641
squirrel monkey	K596D	0.0299
sheep	K596L	0.0623
Bactrian camel	K596M	0.1716
naked mole rat	K596P	0.0673

Supplementary Table S8: Primers for SAMHD1 site directed mutagenesis.

Primer	Sequence
R566Q-F1	CTGTACGCCGCCcaACAGTACTTCGTG
R566Q-R1	CACGAAGTACTGTtgGGCGGCGTACAG
A574S-F1	GTGCAGTGGTGCtCCGACCGGAACTTC
A574S-R1	GAAGTTCCGGTCGGaGCACCACTGCAC
A574L-F1	GTGCAGTGGTGCctCGACCGGAACTTC
A574L-R1	GAAGTTCCGGTCGagGCACCACTGCAC
Q594L-F1	CTGATCACCCCTCtGAAAAAAGAGTGG
Q594L-R1	CCACTCTTTTTTCaGAGGGGTGATCAG
Q594R-F1	CTGATCACCCCTCgGAAAAAAGAGTGG
Q594R-R1	CCACTCTTTTTTCcGAGGGGTGATCAG
K596D-F1	GATCACCCCTCAGAAAgAcGAGTGGAACGACAG
K596D-R1	CTGTCGTTCCACTCgTcTTTCTGAGGGGTGATC
K596P-F1	CTGTCGTTCCACTCTggTTTCTGAGGGGTGATC
K596P-R1	CTGTCGTTCCACTCTggTTTCTGAGGGGTGATC
K596L-F1	GATCACCCCTCAGAAActAGAGTGGAACGACAG
K596L-R1	CTGTCGTTCCACTCTagTTTCTGAGGGGTGATC
K596M-F1	GATCACCCCTCAGAAAAtgGAGTGGAACGACAG
K596M-R1	CTGTCGTTCCACTCcaTTTTCTGAGGGGTGATC

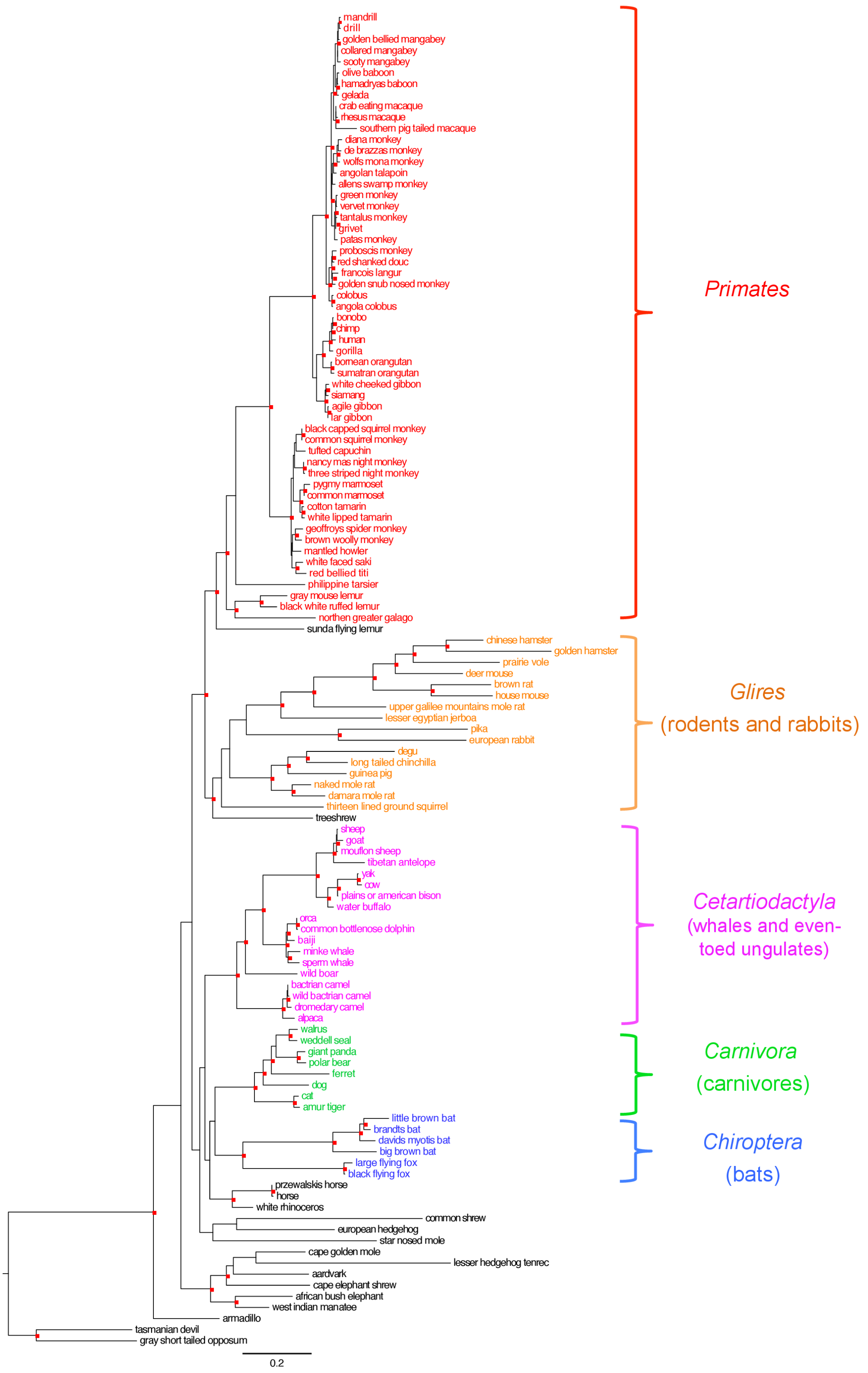

Supplementary Figure S1: Maximum likelihood phylogeny for mammalian SAMHD1, rooted on the division between marsupials and placentals. Red squares mark nodes supported by ≥70% of 1000 non-parametric bootstrap trees. Taxonomic names are given in **Supplementary Table S1**. Branch lengths are expected nucleotide substitutions per codon.

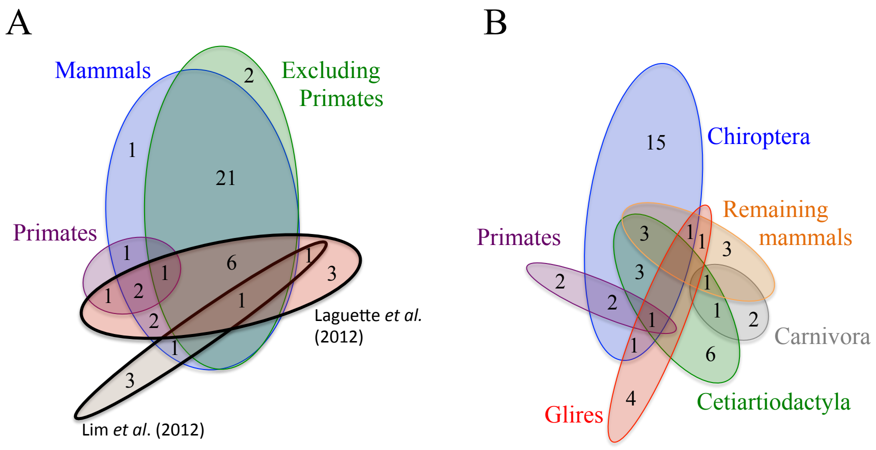

Supplementary Figure S2: Venn diagrams illustrating the numbers of sites identified as under positive selection (BEB posterior probability >0.95) in different analyses of mammalian SAMHD1; compare with **Figure 1** and **Supplementary Table S3**. **(A)** Numbers of sites under positive selection with, without or only primate sequences included and numbers of sites identified in previous studies of primate SAMHD1 (1, 2). **(B)** Numbers of sites under positive selection identified when analysing mammal subgroups individually.

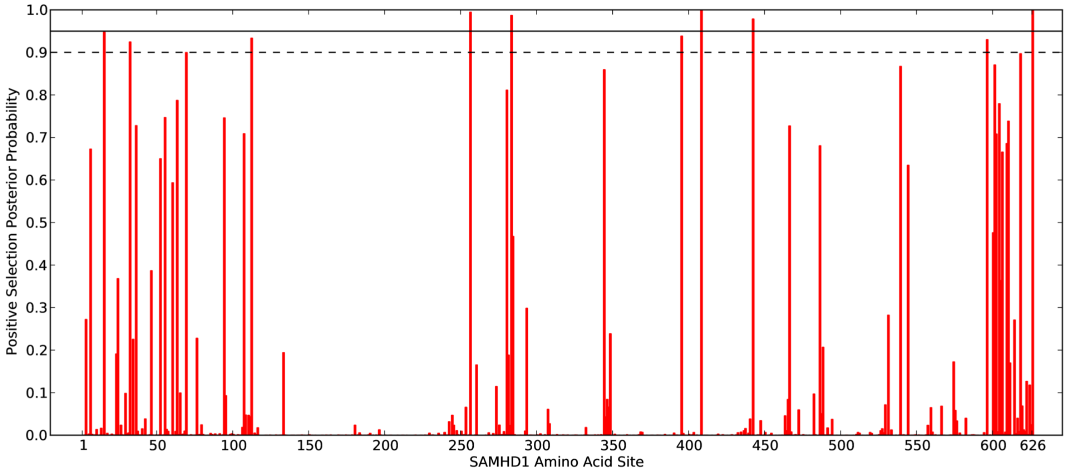

Supplementary Figure S3: Posterior probabilities for Primate SAMHD1 codon/amino acid sites belonging to the positive selection site class in codeml model M8 (7, 8), computed by the Bayes empirical Bayes procedure. The conventional threshold for statistical significance is 0.95, shown as a solid line; the 0.9 level is shown as a dotted line for comparison.

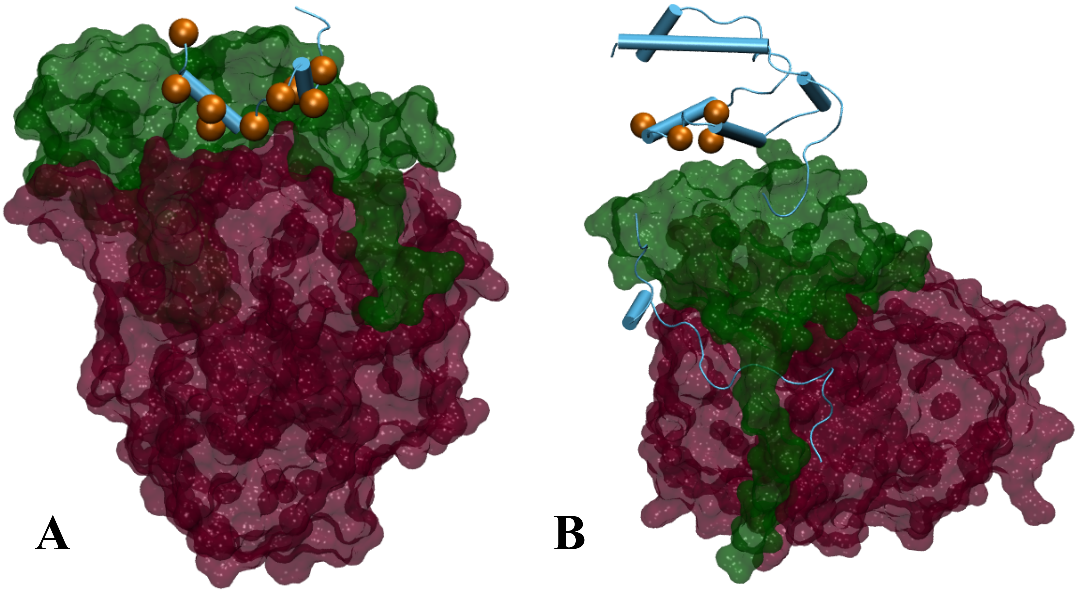

Supplementary Figure S4: Sites identified as under positive selection in mammalian SAMHD1 (α-carbons are shown as orange spheres) in published crystal structures of SAMHD1 (cyan cartoon) in complex with Vpx (green surface) and DCAF1 (dark red surface). **(A)** Human SAMHD1 (residues 606-624), Vpx from SIVsmm and human DCAF1 (PDB 4CC9, ref. (9)); includes 9 of 36 sites identified. **(B)** Mandrill SAMHD1 (residues 1-22, 34-88 and 93-109), SIVmnd-2 Vpx and human DCAF1 (PDB: 5AJA, ref. (10)); includes 4 of 36 sites identified.

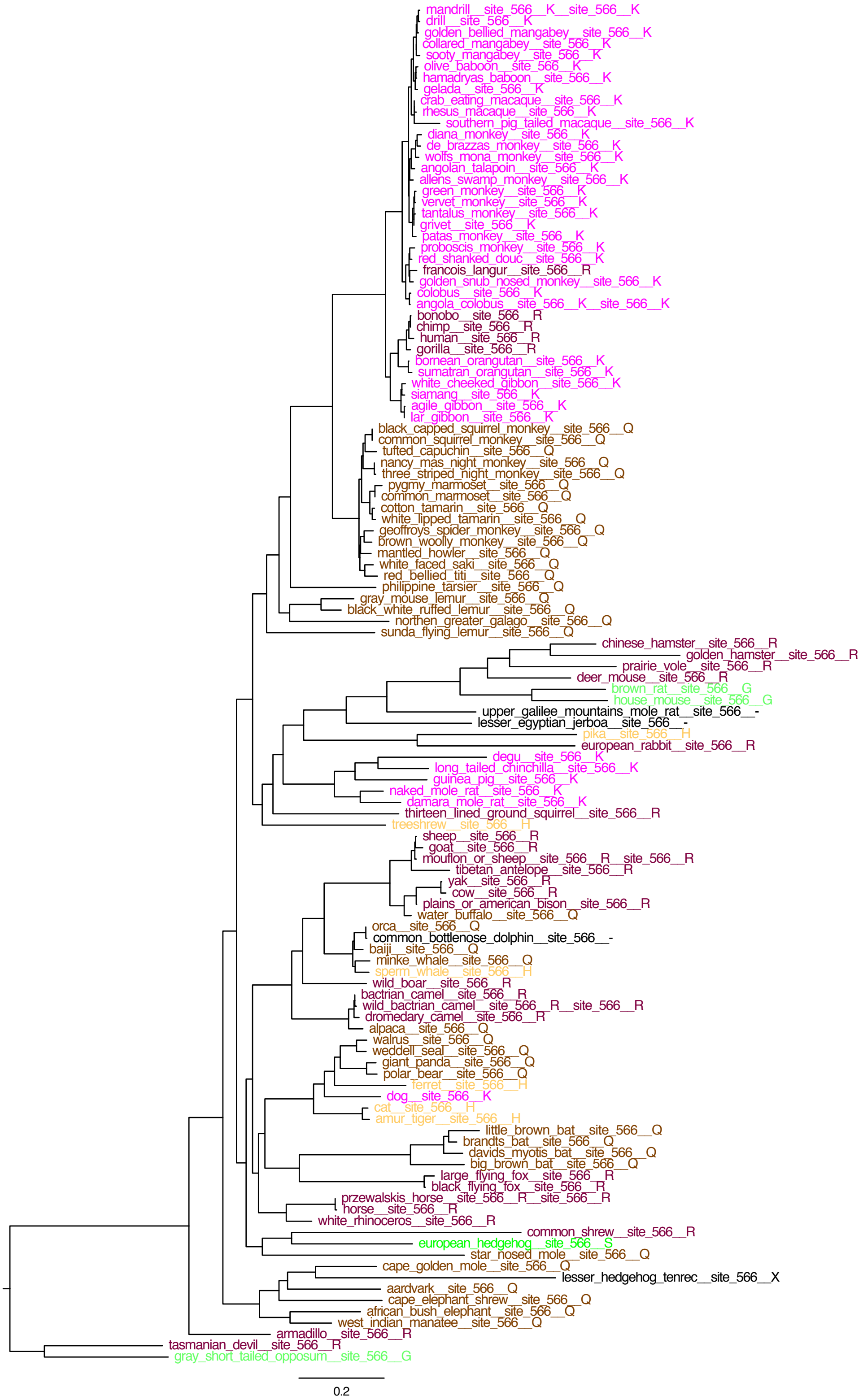

Supplementary Figure S5: Maximum likelihood tree for mammalian SAMHD1, coloured by residues in each sequence at site 566. Topology and node support identical to **Supplementary Figure S1**. Branch lengths are nucleotide substitutions per codon.

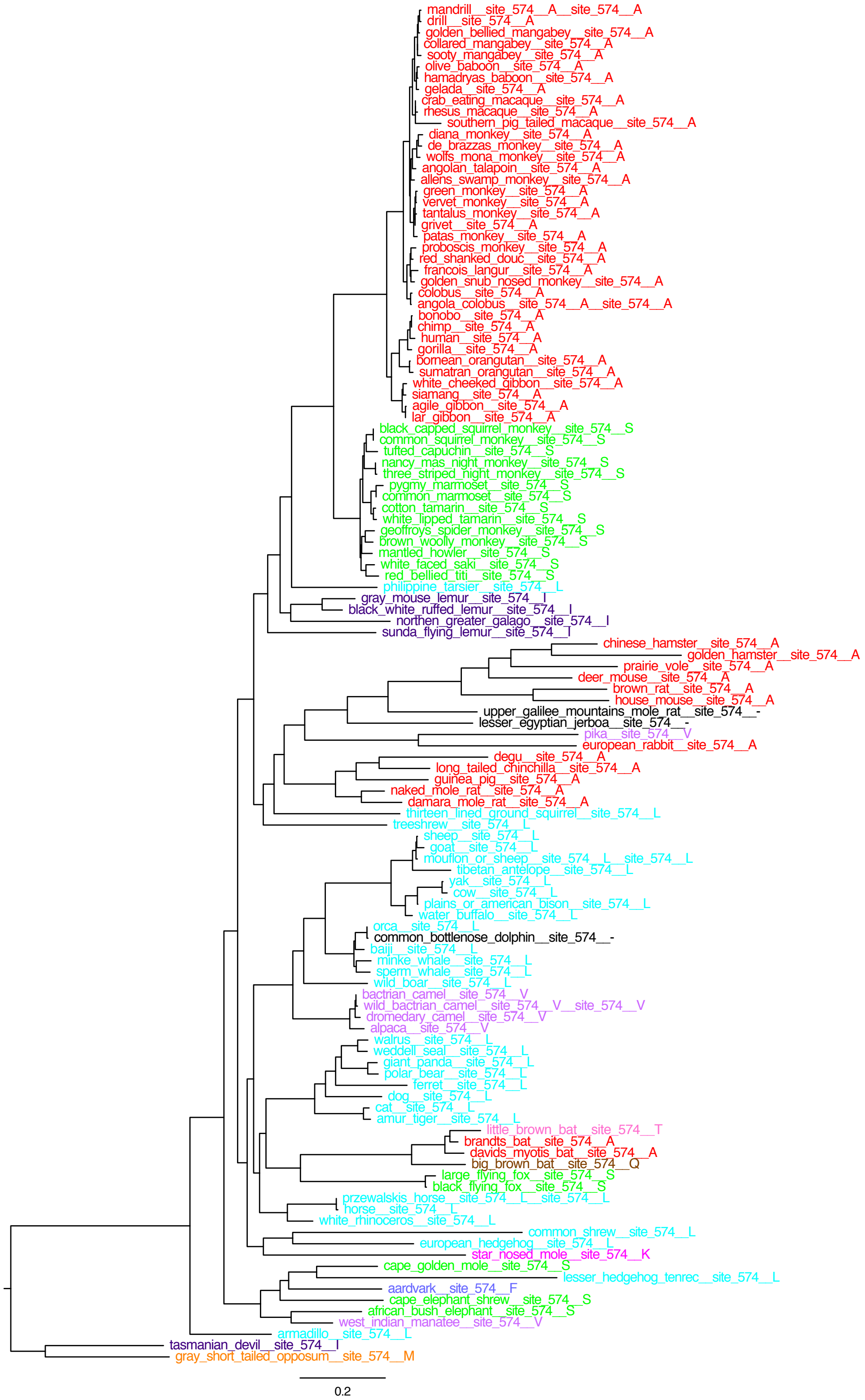

Supplementary Figure S6: Maximum likelihood tree for mammalian SAMHD1, coloured by residues in each sequence at site 574. Topology and node support identical to **Supplementary Figure S1**. Branch lengths are nucleotide substitutions per codon.

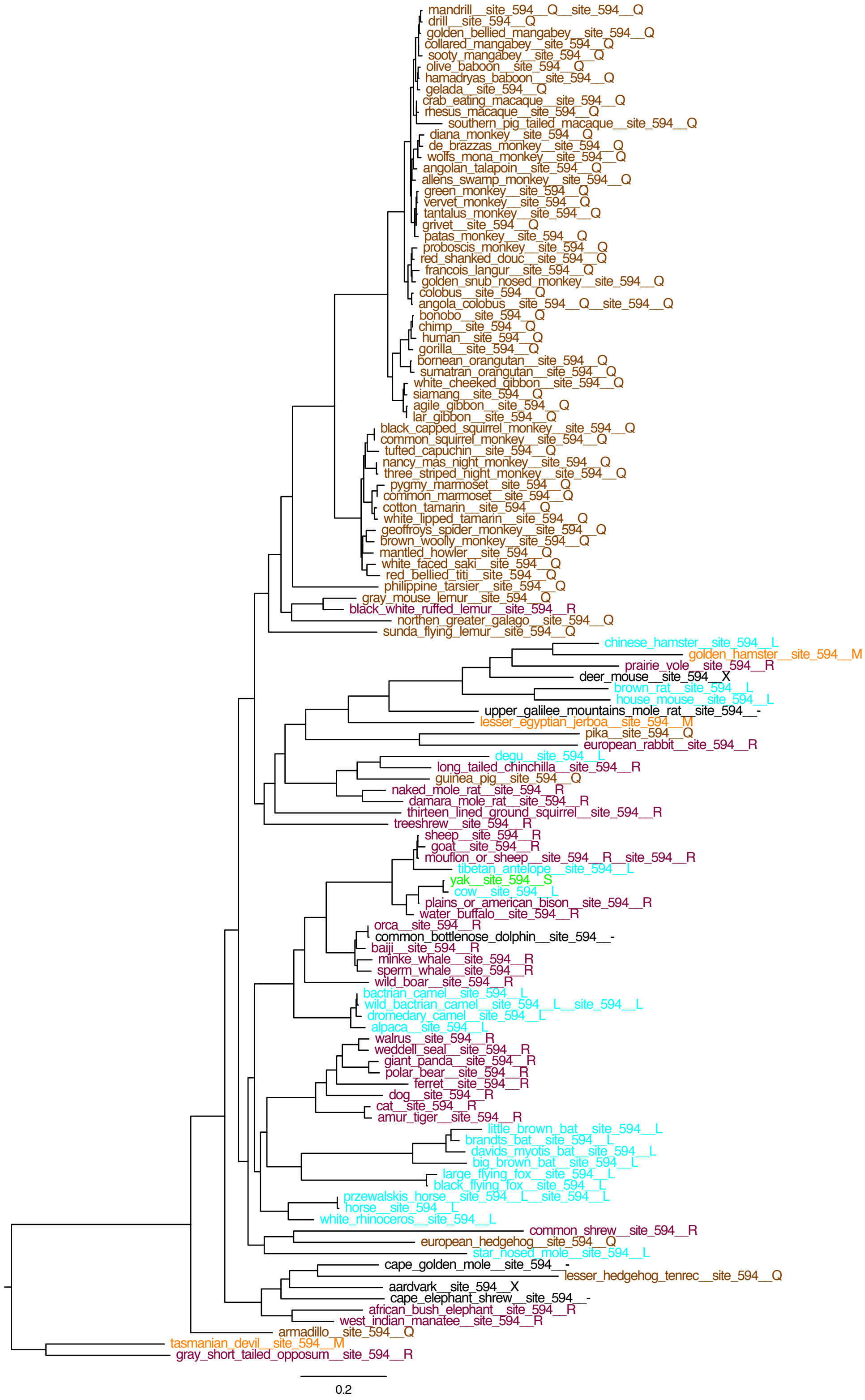

Supplementary Figure S7: Maximum likelihood tree for mammalian SAMHD1, coloured by residues in each sequence at site 594. Topology and node support identical to **Supplementary Figure S1**. Branch lengths are nucleotide substitutions per codon.

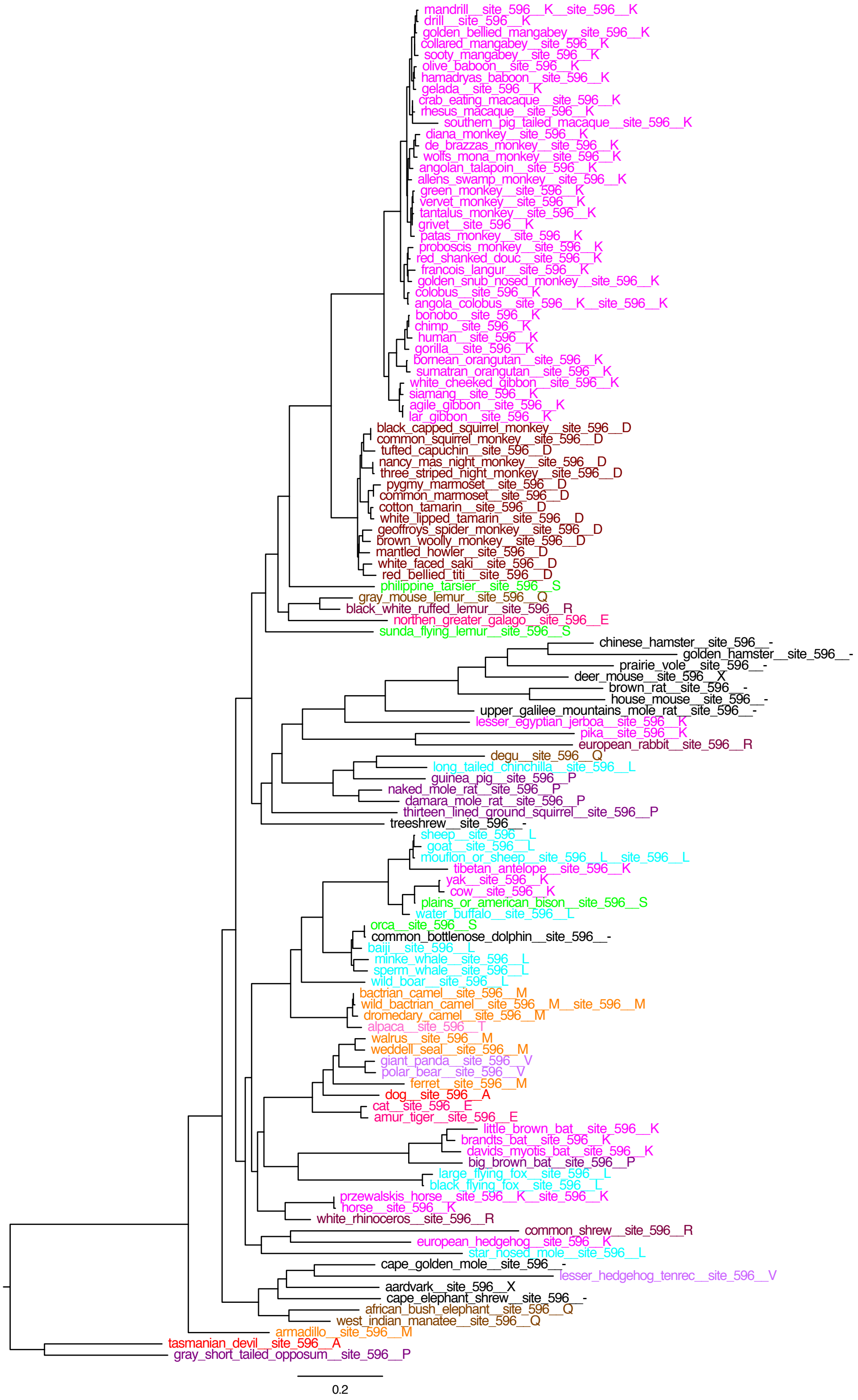

Supplementary Figure S8: Maximum likelihood tree for mammalian SAMHD1, coloured by residues in each sequence at site 596. Topology and node support identical to **Supplementary Figure S1**. Branch lengths are nucleotide substitutions per codon.

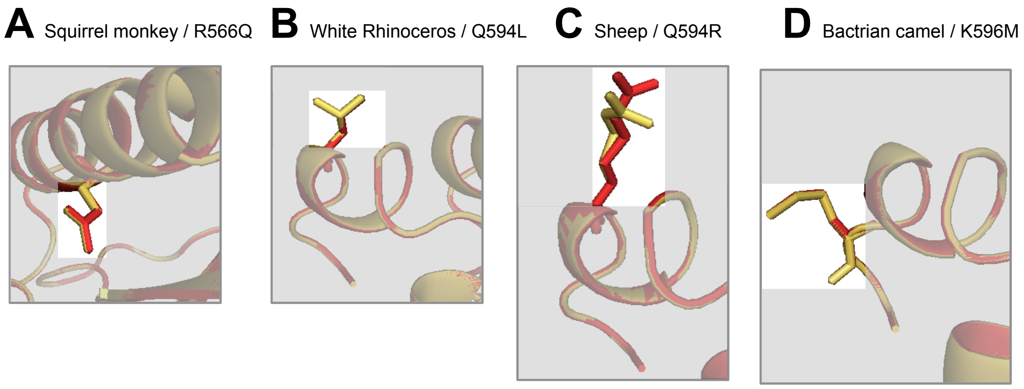

Supplementary Figure S9: SAMHD1 structural models for mammal SAMHD1 sequences (red) and mutant human SAMHD1 sequences (yellow), estimated by SwissModel (see **Supplementary Table S7**) and aligned by minimising the root mean square deviation (RMSD) of homologous Cα atoms. The models are shown superimposed, with backbone residues shown as cartoons and the relevant residues' side chains shown as sticks and highlighted. Regions of mixed colouring represent near total alignment; for all except the sheep/Q594R comparison, the residue side chains are so closely aligned as to be visually indistinguishable from any angle.

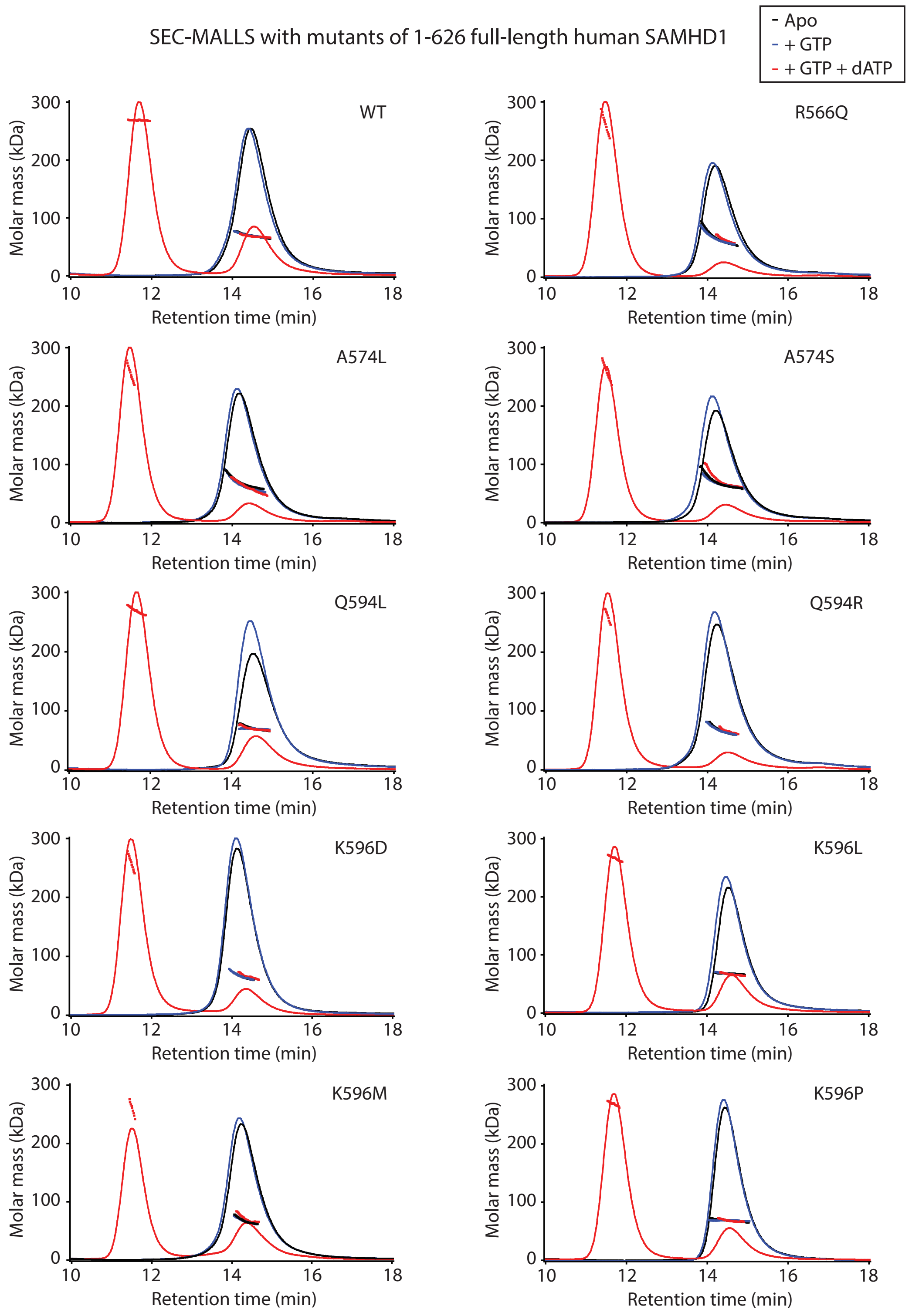

Supplementary Figure S10: The molar mass composition of wild-type (WT) or mutant human SAMHD1 samples analysed by SEC-MALLS. In each panel, solid lines are the dRI chromatograms recorded from the Apo (black), + GTP (Blue) and +GTP and dATP (red) samples. The points, colour coded same as the chromatograms, are the molar mass measured at 1 second intervals throughout the elution of the chromatographic peaks. In each panel, the amino acid substitution identifies the mutant.

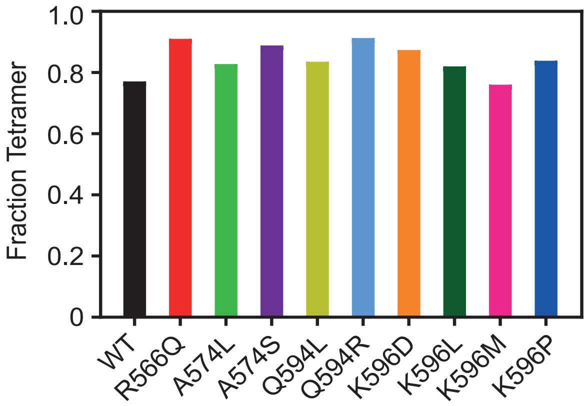

Supplementary Figure S11: Tetramerisation and phosphorylation of human SAMHD1 mutants. Analysis of tetramerisation capacity for each SAMHD1 variant assessed by SEC-MALLS. The bars show the proportion of SAMHD1 tetramer determined by integration of the monomer-dimer and tetramer peaks from the chromatographic data in **Supplementary Figure S10.**

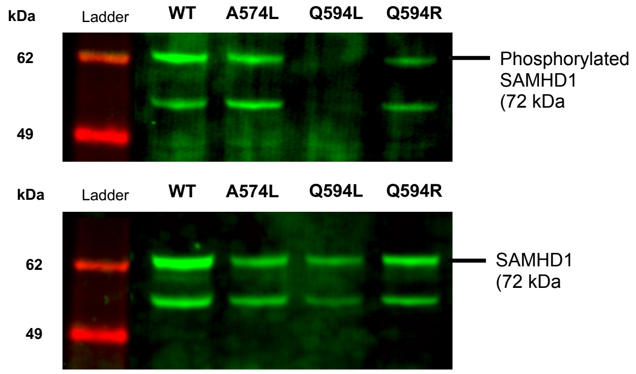

Supplementary Figure S12: Phosphorylation of human SAMHD1 wild-type and mutants. U937 cells were transduced with VLPs co-expressing YFP and different SAMHD1 variants (wild type, lane 1; A574L, lane 2, Q594L, lane 3, and Q594R, lane 4). Equal numbers of YFP positive cells were sorted, lysed and loaded onto SDS-PAGE gels. Phosphorylated SAMHD1 (top panel) and total SAMHD1 (bottom panel) in the samples were detected by immunoblotting with rabbit anti-phosphorylated SAMHD1 and mouse anti-SAMHD1 antibodies, respectively.

Supplementary Information References

1. Laguette N, et al. (2012) Evolutionary and functional analyses of the interaction between the myeloid restriction factor SAMHD1 and the lentiviral Vpx protein. *Cell Host Microbe* 11(2):205–217.
2. Lim ES, et al. (2012) The ability of primate lentiviruses to degrade the monocyte restriction factor SAMHD1 preceded the birth of the viral accessory protein Vpx. *Cell Host Microbe* 11(2):194–204.
3. Biasini M, et al. (2014) SWISS-MODEL: Modelling protein tertiary and quaternary structure using evolutionary information. *Nucleic Acids Res* 42(W1):W252–W258.
4. Ji X, Tang C, Zhao Q, Wang W, Xiong Y (2014) Structural basis of cellular dNTP regulation by SAMHD1. *Proc Natl Acad Sci U S A* 111(41):E4305–E4314.
5. Buzovetsky O, et al. (2018) The SAM domain of mouse SAMHD1 is critical for its activation and regulation. *Nat Commun* 9:411.
6. Holm L, Laakso LM (2016) Dali server update. *Nucleic Acids Res* 44(W1):W351–W355.
7. Yang Z, Nielsen R, Goldman N, Pedersen a M (2000) Codon-substitution models for heterogeneous selection pressure at amino acid sites. *Genetics* 155(1):431–449.
8. Yang Z (2007) PAML 4: phylogenetic analysis by maximum likelihood. *Mol Biol Evol* 24(8):1586–1591.
9. Schwefel D, et al. (2014) Structural basis of lentiviral subversion of a cellular protein degradation pathway. *Nature* 505(7482):234–238.
10. Schwefel D, et al. (2015) Molecular determinants for recognition of divergent SAMHD1 proteins by the lentiviral accessory protein Vpx. *Cell Host Microbe* 17(4):489–499.
